# Supplementary material for: “Are all referrals necessary?” Experiences and perceptions of maternity healthcare providers on emergency intrapartum referrals in Dar es Salaam, Tanzania
Source: PLoS One. 2024 Feb 21;19(2):e0298103. doi: 10.1371/journal.pone.0298103 (PMC10880996; doi:10.1371/journal.pone.0298103)
Supplement: Attachment — Submitted filename: PONE-D-22-34478 reviewed ok (3).pdf [file pone.0298103.s002.pdf]

PLOS ONE

“Are all referrals necessary ?” Experiences and perceptions of maternity healthcare providers on emergency intrapartum referrals in Dar es Salaam, Tanzania.

--Manuscript Draft--

**Manuscript Number:**	PONE-D-22-34478
**Article Type:**	Research Article
**Full Title:**	“Are all referrals necessary ?” Experiences and perceptions of maternity healthcare providers on emergency intrapartum referrals in Dar es Salaam, Tanzania.
**Short Title:**	Experiences and perceptions of maternity healthcare providers on emergency intrapartum referrals.
**Corresponding Author:**	SHEKHA SELEMANI, MMed<br>Muhimbili University of Health and Allied Sciences School of Medicine<br>DAR ES SALAAM, TANZANIA, UNITED REPUBLIC OF
**Keywords:**	emergency intrapartum referrals; maternity healthcare providers; Tanzania
**Abstract:**	Background: A functional referral system is an important component of quality of care. Continuity of care and reduction of maternal morbidity and mortality rely heavily on effective referral system. Capacity building is expected to improve the referral hierarchy but the situation is different. This study aimed at exploring experiences and perceptions of maternity healthcare providers on emergency intrapartum referrals in Dar es Salaam, Tanzania.<br>Methods: An exploratory qualitative study was conducted in Amana regional referral hospital and Muhimbili national hospital. Maternity healthcare providers were purposively recruited using maximum variation sampling. A semi-structured interview guide was used to conduct eleven in-depth interviews. Thematic analysis approach by Braun and Clarke (2006).<br>Results: Two major themes emerged, namely, referral beyond medical indications and poor communication between referring and receiving facilities hindering care. Referrals, according to maternity healthcare professionals, were seen as a way to minimise blame as well as a clinical management tool to prevent difficulties. It was found that, there was limited understanding on skills set among maternity healthcare providers and some had negative perceptions towards performing their responsibilities.<br>Conclusions: Skills gap among maternity healthcare providers at referring hospitals still existed which influenced the decision making and service provision. There was hostility between referring and receiving hospitals. Strategies such as on-going training and capacity building should be emphasized. The referring hospital should only consider referrals as a last resort.
**Order of Authors:**	SHEKHA SELEMANI, MMed
	MICHAEL O MWAKYUSA, MMed
	SELEMANI BASHIRI, MMed
	MANGI M EZEKIEL, Ph.D
	DORKASI L MWAKAWANGA, MSc
	FADHLUN M ALWY AL-BEITY, Ph.D
	ANDREA B PEMBE, Ph.D
**Additional Information:**	
**Question**	**Response**
**Financial Disclosure**<br><br>Enter a financial disclosure statement that describes the sources of funding for the work included in this submission. Review	The author(s) received no specific funding for this work

Powered by Editorial Manager® and ProduXion Manager® from Aries Systems Corporation

the submission guidelines for detailed requirements. View published research articles from *PLOS ONE* for specific examples.<br><br>This statement is required for submission and **will appear in the published article** if the submission is accepted. Please make sure it is accurate.<br><br>**Unfunded studies**<br>Enter: *The author(s) received no specific funding for this work.*<br><br>**Funded studies**<br>Enter a statement with the following details:<br>• Initials of the authors who received each award<br>• Grant numbers awarded to each author<br>• The full name of each funder<br>• URL of each funder website<br>• Did the sponsors or funders play any role in the study design, data collection and analysis, decision to publish, or preparation of the manuscript?<br>• **NO** - Include this sentence at the end of your statement: *The funders had no role in study design, data collection and analysis, decision to publish, or preparation of the manuscript.*<br>• **YES** - Specify the role(s) played.<br><br>* typeset	
**Competing Interests**<br><br>Use the instructions below to enter a competing interest statement for this submission. On behalf of all authors, disclose any competing interests that could be perceived to bias this work—acknowledging all financial support and any other relevant financial or non-financial competing interests.<br><br>This statement is required for submission and **will appear in the published article** if the submission is accepted. Please make sure it is accurate and that any funding sources listed in your Funding Information later in the submission form are also	The authors have declared that no competing interests exist

declared in your Financial Disclosure statement.<br><br>View published research articles from *PLOS ONE* for specific examples.<br><br>**NO authors have competing interests**<br><br>Enter: *The authors have declared that no competing interests exist.*<br><br>**Authors with competing interests**<br><br>Enter competing interest details beginning with this statement:<br><br>*I have read the journal's policy and the authors of this manuscript have the following competing interests: [insert competing interests here]*<br><br>* typeset	
**Ethics Statement**<br><br>Enter an ethics statement for this submission. This statement is required if the study involved:<br><br>• Human participants<br>• Human specimens or tissue<br>• Vertebrate animals or cephalopods<br>• Vertebrate embryos or tissues<br>• Field research<br><br>Write "N/A" if the submission does not require an ethics statement.<br><br>General guidance is provided below. Consult the submission guidelines for detailed instructions. **Make sure that all information entered here is included in the Methods section of the manuscript.**	Ethical clearance was obtained from Muhimbili University of Health and Allied Sciences Institutional Review Board (MUHAS-REC-10-2021-867). The permission to conduct the study was obtained from Director of Clinical Services at Amana Regional Referral Hospital and the Director of Clinical Services at Muhimbili National Hospital. Study participants were informed about study goals and objectives and were asked for permission to digitally audio-record. Information provided by the participant was kept confidential at all times and used for the research purpose. All the voices recorded were deleted after transcription and translation of the information. It was guaranteed that information was treated confidentially, anonymised before publication, and that quotes could not be traced back to individuals. We obtained written consent from all participants participated in the study.

Powered by Editorial Manager® and ProduXion Manager® from Aries Systems Corporation

**Format for specific study types**<br><br>**Human Subject Research (involving human participants and/or tissue)**<br>• Give the name of the institutional review board or ethics committee that approved the study<br>• Include the approval number and/or a statement indicating approval of this research<br>• Indicate the form of consent obtained (written/oral) or the reason that consent was not obtained (e.g. the data were analyzed anonymously)<br><br>**Animal Research (involving vertebrate animals, embryos or tissues)**<br>• Provide the name of the Institutional Animal Care and Use Committee (IACUC) or other relevant ethics board that reviewed the study protocol, and indicate whether they approved this research or granted a formal waiver of ethical approval<br>• Include an approval number if one was obtained<br>• If the study involved *non-human primates*, add *additional details* about animal welfare and steps taken to ameliorate suffering<br>• If anesthesia, euthanasia, or any kind of animal sacrifice is part of the study, include briefly which substances and/or methods were applied<br><br>**Field Research**<br><br>Include the following details if this study involves the collection of plant, animal, or other materials from a natural setting:<br>• Field permit number<br>• Name of the institution or relevant body that granted permission	
**Data Availability**<br><br>Authors are required to make all data underlying the findings described fully available, without restriction, and from the time of publication. PLOS allows rare exceptions to address legal and ethical concerns. See the PLOS Data Policy and FAQ for detailed information.	No - some restrictions will apply

Powered by Editorial Manager® and ProduXion Manager® from Aries Systems Corporation

A Data Availability Statement describing where the data can be found is required at submission. Your answers to this question constitute the Data Availability Statement and **will be published in the article**, if accepted.<br><br>**Important:** Stating 'data available on request from the author' is not sufficient. If your data are only available upon request, select 'No' for the first question and explain your exceptional situation in the text box.<br><br>Do the authors confirm that all data underlying the findings described in their manuscript are fully available without restriction?	
**Describe where the data may be found in full sentences. If you are copying our sample text, replace any instances of XXX with the appropriate details.**<br><br>• If the data are **held or will be held in a public repository**, include URLs, accession numbers or DOIs. If this information will only be available after acceptance, indicate this by ticking the box below. For example: *All XXX files are available from the XXX database (accession number(s) XXX, XXX.).*<br>• If the data are all contained **within the manuscript and/or Supporting Information files**, enter the following: *All relevant data are within the manuscript and its Supporting Information files.*<br>• If neither of these applies but you are able to provide **details of access elsewhere**, with or without limitations, please do so. For example:<br><br>*Data cannot be shared publicly because of [XXX]. Data are available from the XXX Institutional Data Access / Ethics Committee (contact via XXX) for researchers who meet the criteria for access to confidential data.*<br><br>*The data underlying the results presented in the study are available from (include the name of the third party*	Data can not be shared publicly because they contain sensitive participants' informations and participants did not consent to having their interviews shared. Data are available from the Directorate of Research and Publications at the Muhimbili university of health and allied sciences (contact via drp@muhas.ac.tz) for researchers who meet the criteria for access to confidential data.

and contact information or URL).<br>• This text is appropriate if the data are owned by a third party and authors do not have permission to share the data.<br><br>* typeset	
Additional data availability information:	

Powered by Editorial Manager® and ProduXion Manager® from Aries Systems Corporation

Manuscript Click here to access/download;Manuscript;Manuscript Final.doc

"*Are all referrals necessary*?" Experiences and perceptions of maternity healthcare providers on emergency intrapartum referrals in Dar es Salaam, Tanzania.

Shekha Selemani[1*], Michael O. Mwakyusa[1], Selemani Bashiri[1], Mangi M. Ezekiel[2], Dorkasi L. Mwakawanga[3], Fadhlun M. Alwy Al-beity[1], and Andrea B. Pembe[1]

Authors Affiliations

[1]Department of Obstetrics and Gynecology, Muhimbili University of Health and Allied Sciences, Dar es Salaam, Tanzania.

[2]Department of Behavioral Sciences, Muhimbili University of Health and Allied Sciences, Dar es Salaam, Tanzania.

[3]Department of Community Health Nursing, Muhimbili University of Health and Allied Sciences, Dar es Salaam, Tanzania.

***Correspondence:** Shekha Selemani; Tel: +255782205707

E-mail: shekha_alinisi@yahoo.com

ORCID iD: https://orcid.org/0000-0002-8965-2827

Abstract

Background: A functional referral system is an important component of quality of care. Continuity of care and reduction of maternal morbidity and mortality rely heavily on effective referral system. Capacity building is expected to improve the referral hierarchy but the situation is different. This study aimed at exploring experiences and perceptions of maternity healthcare providers on emergency intrapartum referrals in Dar es Salaam, Tanzania.

Methods: An exploratory qualitative study was conducted in Amana regional referral hospital and Muhimbili national hospital. Maternity healthcare providers were purposively recruited using maximum variation sampling. A semi-structured interview guide was used to conduct eleven in-depth interviews. Thematic analysis approach by Braun and Clarke (2006).

Results: Two major themes emerged, namely, referral beyond medical indications and poor communication between referring and receiving facilities hindering care. Referrals, according to maternity healthcare professionals, were seen as a way to minimise blame as well as a clinical management tool to prevent difficulties. It was found that, there was limited understanding on skills set among maternity healthcare providers and some had negative perceptions towards performing their responsibilities.

Conclusions: Skills gap among maternity healthcare providers at referring hospitals still existed which influenced the decision making and service provision. There was hostility between referring and receiving hospitals. Strategies such as on-going training and capacity building should be emphasized. The referring hospital should only consider referrals as a last resort.

Keywords: Emergency intrapartum referrals, maternity healthcare providers, Tanzania

Introduction

A functional referral is an important component of World Health Organization (WHO) quality of care (fig 1) for maternal and newborn health [1, 2]. In 2015, The United Nation launched Sustainable Development Goals target 3.1 which aimed at reducing global maternal mortality ratio to less than 70 per 100,000 live births by 2030 [3] . An efficient referral system is central and essential for continuity of care, and the reduction of maternal morbidity and mortality [4]. By maximizing the limited health facility capacities, a good and functional referral system increases the efficiency of the health system.

Timely referral to an appropriate health facility for addressing maternity needs is a key indicator of a functional health system [5]. Multi-sectorial collaboration and well-coordinated levels of care, linking communities with essential maternal and new-born care is a necessary component of a high-quality referral system [6]. In low and low-middle income countries, emergency intrapartum referrals service is still a major challenge [7]. In Tanzania, majority of referrals within Dar es Salaam region to MNH have originated from public health facilities. The most common reasons for referral include lack of expertise, lack of necessary equipment; other reasons were lack of drugs and physical space [8].

Referrals are referred to as safest option for health care professionals to prevent the devastating consequences on the maternal and fetal outcomes [9]. However, referrals are often done to avoid complications and deaths to occur within their own facility. The woman referred has been described as “not my case” [9] . A study conducted in rural Zambia found that there was minimal consensus on decision-making for specific diseases because no suitable referral strategy was used for each problem [10]. Moreover, excessive requests for delivery care services at health facilities, puts health providers under pressure, which may influence the increase in number of referrals to tertiary hospitals [11]. A qualitative study done by Afari in Ghana showed that maternity care providers perceived some challenges related to poor quality within their designated skill set which led them to refer patients to tertiary hospitals [12] . A lack of pre-referral communication, high number of patients at referring hospital and the absence of feedback all contribute to an increase in intrapartum referrals and a dysfunctional referral system [13–15].

Studies have been done on perceptions of women and maternal care providers on effectiveness and quality of maternal referral system and obstetric care in referral hospitals. However, all these studies were conducted in rural settings of Tanzania [4, 16, 17]. Little is known about perceptions and experiences of maternity care providers on emergency intrapartum referrals in urban settings. Therefore, this study reports the perceptions and experiences of maternity care providers on emergency intrapartum referrals at a regional referral hospital and a tertiary Hospital in Dar es Salaam City, Tanzania.

Fig 1: WHO Quality of Care Framework for maternal and newborn health

A Conceptual Framework

Fig 2: A conceptual framework illustration

The illustration above shows constructs that may influence perception and experience of maternity care providers on emergency intrapartum referrals. Since we were unable to find an existing framework for examining the perceptions and experiences of maternity care professionals about emergency intrapartum referrals, we adapted a framework from a relevant study in order to develop a framework for this study [18]. The framework of this study demonstrates that Standard Operating Procedures availability and usage, patients' demands and satisfaction, patients' attitudes towards maternity care providers, referrals (safest option for healthcare providers), poor skill sets and working environment are among the constructs that may influence perceptions of maternity healthcare providers and resulting into increment of emergency intrapartum referrals. Competence among maternity healthcare providers and minimal training, working environment such as long working hours, heavy workload, weak supervision and communication with co-workers, and insufficient salaries can contribute to a negative experience among workers, which may also result in a referral of a patient. As a result of a particular experience, maternity healthcare providers at the receiving hospital held a particular view about referrals from a particular facility.

Materials and Methods

Study design and setting

This was an explorative qualitative study using in-depth interviews to explore maternity healthcare provider's perceptions and experiences on emergency intrapartum referrals in Dar es Salaam region. The Tanzania health system is a pyramidal structure where a higher hospital level supports a network of lower-level facilities [19]. A district hospital serves as first referral level for their immediate lower-level facilities health centres and dispensaries. At this level, patients in need of advance services are transferred to the secondary level hospitals at regional level then at the tertiary level which include zonal, specialized and National Hospital. The study was done in Dar es Salaam, the largest city with highest population density in Tanzania of about 5,383,728 [20]. In Tanzania, the healthcare referral system is organized in hierarchal manner: the district hospitals refer patients to regional health facilities that refer patient to the tertiary level health facilities. There are national guideline and protocols that describe the referral process, however these are not widely known or followed. This study was done in two referral levels, Amana regional referral hospital, a secondary-level facility, and Muhimbili national hospital, a tertiary-level facility. The two facilities are both urban and have similar but different characteristics as seen in **Table 1**.

Table 1. Similarities and differences between the two health facilities in the study

**Characteristics**	**Amana Regional Referral Hospital**	**Muhimbili National Hospital**
Referral level	Secondary level, receiving from district hospitals and large health centres	Tertiary level, receive from three secondary level hospitals, and from nearby Pwani region hospitals such as Bagamoyo, Mkuranga and Kisarawe
Infrastructure	Separate maternity building with antenatal, postnatal, neonatal, labor ward with six delivery beds<br><br>No separate Intensive Care Unit or High dependence unit<br><br>Dedicate obstetric theatre with two beds within the maternity building	Separate maternity building with antenatal, postnatal, neonatal, labor ward with six delivery beds<br><br>Has a dedicated Intensive Care Unit and High dependence unit within the maternity building<br>Dedicate obstetric theatre with four beds adjacent to maternity building
Human resource	Seven medical specialists (obstetric/gynecologist), six registrars and no residents<br><br>65 midwives of different level of training (certificate to master's level)<br><br>13 Anesthetist staffs	Twenty-six medical specialists (obstetric/gynecologist and consultants), sixteen registrars and more than 30 residents who are medical doctors specializing in obstetrics/gynecology.<br><br>159 midwives of different level training (diploma to master's level)<br><br>8 Anesthetist staffs per shift in obstetric theatre
Daily delivery load	39-45 total deliveries per day	25-35 total deliveries per day
Patient flow	Direct from home and referrals from within the district.	Direct from home and referrals from Primary and secondary level facilities<br><br>Muhimbili National Hospital also has an Intermural Private Practice.

The referral process from Amana to Muhimbili National Hospital

At Amana: Once a referral decision has been made, the specialist on duty or head of department is consulted to examine the patient and authorize the referral. The nurse supervisor contacts Muhimbili National Hospital, the receiving facility, via telephone. They describe the patients' diagnosis, condition, reason for referral and management given. The supervisor organizes the ambulance, and a nurse escorts the woman to the higher-level facility. Private cars are used to transport patients when the ambulance is not available due to lack of fuel or an absence of a driver. There is a cost associated with renting a private ambulance.

At MNH: the mobile phone stays with the specialist on duty. The specialist carries a dialogue with Amana, provides advice on further interventions, accepts or redirects the referral to Mloganzila hospital, which is another branch of MNH.

When a woman is referred with an intrapartum emergency, she is received at MNH labor ward, where initial management including resuscitations is done. The on-call medical team reassesses and decides on patient disposal, including whether she needs further consultations with senior doctors, interdepartmental consultations, or surgical management.

Study participants and recruitment

Purposive sampling technique was used to recruit maternity care providers of different cadres and levels of training as well as maternity ward administrators. The administrators were maternity wards in-charge, Medical Officer in charge of the labor ward, Head of department from the two facilities. These groups of participants were involved in managing emergency obstetric cases and implementing emergency intrapartum referral policies. The researcher requested maternity in charges in the two health facilities to provide a list of all maternity care providers. Purposive selection was based on three years and above of experience in the department to capture an experience on referral system, cadre, and their roles in the referral process. Eleven participants including doctors, midwives, and administrators (in-charge of maternity wards, head of department) working at Amana regional referral hospital and MNH participated in the study. The identified participants were contacted by the researchers either in person or by phone to establish rapport and to set a day and time for an appointment to participate in interviews.

Data collection

The data were collected in the two hospitals using semi- structured interview guide which was prepared in English and then translated into Kiswahili language which was used for conducting interviews. The guide contained main questions and probes to give a chance to the participant to provide more responses or clarification to what has been said. The first author conducted interviews with the assistance of two research assistants who took notes and audio recorded the interviews. Research assistants had sociology training and were well conversed with qualitative data collection. Written informed consent was obtained from each participant for taking part in the interview and for audio recording. Interview procedures were explained, and each participant was informed that his/her participation is voluntarily and may choose not to answer some of the questions or stop the interview at any time. The in-depth interviews took place in a quiet private room within the respective facility at their convenient time suggested by them. All interviews were conducted in the Kiswahili, the native language spoken comfortably by researchers and study participants. All interviews were audio-recorded and lasted between 45 and 60 minutes. The interviews stopped after data saturation was observed that is when there was no new information being obtained from the study participants and the redundancy had been achieved.

Data processing and analysis

Thematic analysis approach according to Braun and Clarke which involves six steps guided the analysis of data [21]. The analysis was done concurrently with data collection. The analysis began after completion of the first two individual interviews. Audio recordings of the interviews were transcribed verbatim then translated into English by data collection team. The translated transcripts were read through several times to become familiar with the data and to get understanding of the participants accounts. Two researchers coded the first transcript independently and then discussed and reached consensus on the use of codes. Coding was done manually by hand. A list of potential and initial codes reflected the predetermined themes were created. Then sub-themes and themes were then generated from the general list of codes created through comparisons. Lastly, themes were reviewed and discussed by the researchers, discrepancies were noted and identified themes were finalized.

Ethical approval and consent to participate

Ethical clearance was obtained from Muhimbili University of Health and Allied Sciences Institutional Review Board (MUHAS-REC-10-2021-867). The permission to conduct the study was obtained from Director of Clinical Services at Amana Regional Referral Hospital and the Director of Clinical Services at Muhimbili National Hospital. Study participants were informed about study goals and objectives and were asked for permission to digitally audio-record. Information provided by the participant was kept confidential at all times and used for the research purpose. All the voices recorded were deleted after transcription and translation of the information. It was guaranteed that information was treated confidentially, anonymised before publication, and that quotes could not be traced back to individuals. We obtained written consent from all participants participated in the study.

Results

We interviewed 11 participants with age range 29-56 years. Average age of the participants was 40 years, where 45% were female and 55% were male. Among them, five were midwives, two registrars, three obstetrics and gynecologist and one administrator. All of them had a working experience of at least three years.

Two main themes emerged from the analysis process each with 3 to 6 sub-themes: (1) Referral beyond medical indications (2) Limited communication between referring and receiving facilities. **See table 2.**

Table 2: Themes and Sub-themes

**Themes**	**Sub-Themes**
Referral beyond medical indications	- The process of referral<br>- Lack of basic knowledge and skills among MCP<br>- Referrals due to mismatch between delivery load and available facilities<br>- Referrals due to challenges in infrastructures<br>- Referrals to avoid blames<br>- Lack of motivation
Limited communication between referring and receiving facilities	- MCP in receiving facilities perceive MCP in referring facility as incompetent and irresponsible<br>- Harsh treatment to an escort nurse<br>- Lack of constructive feedback to the referring facilities

Theme 1. Referral beyond medical indications

The process of referral

Most of the referrals made were emergency intrapartum referrals. Prior referral, standard operation procedures was reported to be not always followed as patients may be referred without the required pre-referral management or proper documentation in the referral form. During the night, there was increase in number of referrals compared to during the day, and it was reported there was poor supervision to junior doctors during the night which influenced the decision making and referrals at late stage. A participant from receiving hospital quoted saying,

> *"…you find a patient brought with an emergency condition having 18-20 gauges instead of big cannula or was resuscitated with 5 litres of fluid but came with no catheter in situ and the patient is bleeding, you consider this as being lazy or they don't know their work…" (Administrator, receiving hospital)*

It was responded with participant from referring hospital was quoted saying,

> *"… sometimes we receive many patients at once who need to be referred, so following SOPs before referral might be difficulty, but in many cases, referrals must be issued out by the doctor upon agreement, and a referral form will be signed" (Overall in-charge, referring hospital).*

Lack of basic knowledge and skills among MCP

Inadequate knowledge and skills among maternal care providers was found to exist, where some were incompetent in performing their responsibilities and junior doctors' failure to consult to their seniors ended up making wrong decisions and consequently causing delay in provision of management to the patient. A participant was quoted saying,

> *"… Most of the time there is a gap, you find an intern doctor can't decide and still does not consult. At the end, decisions for referral are made very late" (Specialist, referring hospital)*

A participant from receiving hospital was quoted saying, *"…as i was working in labour ward, there was a patient referred with a diagnosis of previous scar with impending rupture, but after our investigation she was found to have umbilical hernia…" (Midwife, receiving hospital)*

Referrals due to mismatch between delivery load and available facilities

Overwhelming of patients at referring hospitals was perceived to be the influencer of giving referrals. The fact that the hospital's infrastructure, such as its operating rooms, neonatal and maternal intensive care units, and blood and transfusion product availability, prevents it from functioning as a referral hospital, which discourages it from providing referrals as a means of receiving assistance. A participant was reported,

"… Right now, we have a big number of patients that we receive who are really emergencies but the environment itself in this hospital is not suitable to receive all of them…" (Specialist, referring hospital)

Another participant was quoted saying,

"…you find a patient was referred from Mbagala Rangi Tatu hospital to Temeke hospital and straight was referred to Muhimbili hospital, that shows there was high number of patients at Temeke that they couldn't manage that patient from Mbagala…" (Midwife, receiving hospital).

Referrals due to challenges in infrastructures

There was unavailability of infrastructures such as intensive care units, high dependency units, enough operating rooms, blood supply for transfusion and an ambulance. When more than one emergency arises, then referral had to issue. And because of availability of one ambulance, then they prefer to call for a referral at upanga compared to mloganzila because of the distance factor. To drive this point home, a participant quoted saying,

"Yesterday when I was on duty, there was a PPH patient and another ectopic patient and both needed quick treatment, so I was forced to go into the theatre with the PPH patient and I referred the Ectopic to another hospital. But if there were enough rooms but also health workers, we could serve both patients at the same time" (Registrar, referring hospital).

Another participant from receiving hospital said,

"A referral was made early for a woman with fetal distress or two prior scars in labor, but you discover the patient is brought 6 or 8 hours later when you inquire and learn about the difficulties in finding an ambulance or an escort nurse who must cycle between more than two hospitals." (Midwife, receiving hospital)

Referrals to avoid blames

When a patient or patient's relative is medical personnel or has influence from the government, they often demand to get a referral even though there is no genuine reason. Most of these challenges are facing hospital administrators who must bear the burden of receiving the phone calls demanding a referral to a certain patient.

A participant was quoted saying,

> *"…But when a relative wants or is in favour of getting a referral to her relative and then you decide to retain a patient, when things go wrong, you can take the blame" (Administrator, referring hospital).*

Another participant adding by saying, *"…we sometimes give referral to the patient whom we could manage them here, only to avoid delays and blames…" (Registrar, referring hospital.*

Lack of motivation

It was reported motivation of the providers was lowered when not receiving their allowances on time and end up not performing well their responsibilities. Some doctors may have a desire of not managing all the patients who comes at referring hospital during his/her duty. He would choose to see some of the patients and refer other patients so that he/she could rest. Either participant reported other doctors work as a routine by not following SOPs and make referral without genuine reason. A participant was quoted saying,

> *"We receive some referrals without an informing call and no convincing reason of referral, some doctors will just refer the patient knowing when the patient reached at receiving hospital will never be rejected and taken back to our hospital" (registrar, receiving hospital).*

Theme 2. Limited communication between referring and receiving facilities

When the woman referred with an intrapartum emergency, she is received at MNH labor ward where an initial management including resuscitations is initiated. The on-call medical team reassesses and decides on patient disposal: whether she needs further consultations with senior doctors need for inter-departmental consultations, or surgical management.

MCP in receiving facilities perceive MCP in referring facility as incompetent and irresponsible

On the process of giving a referral, participants at referring hospitals noted a communication problem with receiving hospital, when they make a call for referral, sometimes they get declined with negative responses and be required to send the patient to another hospital, and because of distance patients ended up with adverse outcome. Participant was quoted saying,

> *"… You end up asking yourself, 'did I come with the patient from my house to Amana? So, we end up taking her to Mloganzila (name of the hospital) …" (Administrator, referring hospital).*

A participant from receiving hospital had a different perception on the clients load and was quoted saying,

> *" … maybe they receive many patients, and they fail attend all of them. You find there is no genuine reason to refer them. They call for referral of two patients and they bring 4 of them..." (Overall in-charge, receiving hospital).*

Harsh treatment to an escort nurse

Escorted nurse who arrived with the patient at receiving hospital could be less informed about the condition of the patient and the referral form was not filled accordingly. The predicament led the maternity staff at the receiving hospital to act hostilely against the escort nurses by presuming they would not do their duties as requested. A participant reported,

> *" … I was called to bring this patient while I was in another ward, it can be challenging to know all the patient's information, especially if there are multiple patients. And when*

you get to the hospital that will be receiving you, the staff will start acting haughtily and asking questions as if we don't want to fulfill our obligations, even though that isn't the case…" (Midwife, referring hospital).

Lack of constructive feedback to the referring facilities

Participants reported that, feedback from the receiving hospital was not always given. Sometimes it was given in a form of report which tends tend up to the level of administration and not reaching to the Maternal care providers. For mismanaged patients who ended up in maternal death, feedbacks were given through phone call.

"I was thinking there should be a procedure of sending feedbacks to the maternal care providers on the patients they referred and their outcome, we do write weekly report, but it ended up at managerial level and not reaching to the providers" (Midwife, receiving hospital)

A participant from referring hospital had this to say,

"*…They hardly ever bring us feedback or let us, the providers, receive it. Feedback rarely reaches the service providers …" (Registrar, referring hospital)*

Discussion

This study explored the perceptions and experiences of maternity care providers on emergency intrapartum referrals in a regional referral hospital and Muhimbili National Hospital in Dar es Salaam, Tanzania. In response to our in-depth interviews, the referrals beyond medical indications such as referring a patient to avoid blames and deaths to occur at a particular health facility and limited communication between a referring and receiving health facility such as lacking constructive feedback and use of harsh language to escort health personnel were found to be the main findings.

Our study found out that decision-making for provision of referral was influenced by a high demand of patients' needs for receiving quality delivery services. It happened when a patient is health personnel, or has an influence from the maternal care providers or from the government.

Since every one wish to receive the best care and most of the patients regards the national hospital has the best care that's why they demanded to be referred even without a genuine reason. Similar findings on patients pressure towards delivery of services were reported from studies done in Indonesia and Tanzania [9, 11], where it showed providers perceived patients to be too demanding during referral process. Furthermore, the views of the patients toward the services offered by the hospital put them under pressure and have an impact on the referrals made. The delivery of the service depends heavily on the patient and provider having a good relationship.

Intrapartum period is very crucial period where close care and monitoring needed to be done so as to ensure both mother and the baby are safe. In an attempt of meeting patient's demands and avoiding adverse outcome, maternal care providers tend to refer patients even without clear reason so as to avoid blames and delays and sometimes made a referral without pre-referral communication [11]. Similar findings on referral as a means to avoid delays and blames was found from the study done in Sierra Leone [12] showed some referrals were done to avoid complications or maternal death occurring at their facility. Maternal care providers may provide referrals to a patient so as to avoid blames if any adverse outcome happened or after receiving negative feedbacks. This could be explained by fear of destroying their reputation as a MCPs as well as a hospital if any bad outcome happened.

The unfriendly working environment included unavailability of intensive care units and high dependency units for adults and neonates, deficit of enough supply of blood for transfusion, less number of operating rooms, little number of staffs and not paying their allowances on time had made them lower their motivation at work since they can't manage the patient until the end. Similar studies reporting shortage of staffs being a major challenge that influence referrals [11, 16, 22]. A study in Ghana, Ethiopia and Indonesia [11, 16, 23], also showed similar findings on unavailability of sufficient infrastructure and number of staffs where referrals contributed by deficiencies in working condition and environment of maternal care providers contributed a lot of stress, frustrations, fatigue and poor job satisfactions and loss of morally. When providers are not paid their allowances on time, it lowers their morale on working to the high level. This could be due to the government had upgrade these hospitals to be referral hospitals where there will be increase in number of patients and work load, but the working environment remain the same with

the same number of staffs. This brought a challenge of increasing number of referrals to the tertiary hospital because of being overwhelmed.

From our study some of the providers lack skills in managing patients, could be newly employed providers or interns or those who are resistant to learn caused delaying in provision of proper management or wrong decision in referring the patient. This could be explained as there was lack of proper supervision from their seniors who were not available especially at night. Similar studies reported showed inadequate training, which lead to lack of skills and poor remuneration facilitates maternal care providers to refer patients to another facility [18]. Another studies showed [12, 24] low quality within their designated skill set made influence providers in referring intrapartum women. Some colleagues were not competent in making diagnosis to a patient since he/she had little experience at work compared to senior provider; hence tend to refer more patients because of luck of skills and confusion on clinical criteria for referral [23, 25]. The similarity could be because of lack of motivation to the seniors due to not being paid their allowances on time, so they will only work during the day and go find other part time jobs to raise their income.

Provision of feedback provide an opportunity for the providers to learn where they did wrong or what was a proper way of handling different cases and so improve quality of services. From our study feedback was not given all the time, there was no formal way of giving out feedbacks. This could be the weakness from the administration, where staffs responsible were not performing their duty or raising challenges if they had any on delivering feedbacks. Studies done highlighted on unavailability of regular feedback provided to the referring facilities and inconsistent use of feedbacks forms instead only complaints have been given on what was not appropriately done or was expected to be done [14, 26]. Other studies reporting similar findings on feedback of referrals here it showed referring facilities were not always given feedbacks, and sometimes feedbacks given to the women or other close family members [6]. Lack of proper referral guidelines had made no uniformity in delivering of services especially giving feedbacks which is a barrier to a functional referral system [15].

Methodological considerations

Trustworthiness in a qualitative study is attained when the findings of such a study are worth believing [27]. Four criteria as explained by Shenton AK, et al. were used to assess the trustworthiness in our study; credibility, dependability, transferability, and conformability [28]. Use of an In-depth Interview using maximum variation of experiences and cadres contributed to the credibility of our study findings. The review and examining the research process showed dependability, deriving the interpretations and highlighted every step of data analysis and transferability by providing clear think descriptions of all aspects that allows reader to judge the applicability.

The study achieved rich data by involving majority of maternity HCPs involved in provision of maternal care and referrals in the facilities that enabled giving a clear picture of what is happening in referral system between referring facilities. Furthermore, it provided deeper views of providers concerning intrapartum referrals to identify where the gap is and what could be better way to work on it. Despite all these strengths, the study had some limitations. Exploring only perceptions and experiences of maternal care providers narrowed the extend of data to be gathered as there are many other angles related to emergency intrapartum referrals which could have been left out related to mothers and their relatives' views on the referrals.

Conclusion

Our study found out that there was a referral hostility between referring and receiving hospital. Skills gap among maternal care providers at referring hospitals still existed which influence the decision making and provision of quality services. Referral should be regarded as the last option after the patient has been managed to the best of knowledge at referring hospital, and pre-referral management should be provided prior referral. Strategies to strengthen communication between the hospitals are warranted. These include on-going trainings should be emphasized. Further studies on experiences of women and their relatives on referral services should be done, to get a clear picture of where we are lacking and what need to be done.

Acknowledgements

The authors acknowledge the support received from all the participants in this study. We also thank Amana Regional Referral Hospital and Muhimbili National Hospital for granting access to use their facilities in this study.

Authors' contributions

SS conceptualized the study, conducted data collection, analysis, and wrote the original draft. ABP and MME aided the conceptualization, analysis and supervised the writing. DLM and FAA participated in the analysis, writing, and reviewing the manuscript. MOM and SB edited and reviewed the manuscript. All authors reviewed and approved the final manuscript.

Conflict of interest

The authors declare that they have no conflicting interests.

Data availability

Data cannot be shared publicly because they contain sensitive participants' information and participants did not consent to having their interviews shared. Data are available from the Directorate of Research and Publications at the Muhimbili university of health and allied sciences (contact via drp@muhas.ac.tz) for researchers who meet the criteria for access to confidential data.

Funding

The author (s) received no specific funding for this work.

References

1. WHO. Quality, Equity, Dignity: A Network for Improving Quality of Care for Maternal, Newborn and Child Health. 2017; February:14–6.

2. TDHS. Tanzania Demorgraphic and Health Survey Indicator Survey (TDHS-MIS) 2015-2016. Dar es Salaam, Tanzania, Rockville, Maryland, USA MoHCDGEC, MoH, NBS, OCGS, ICF. 2016;1:1–630.

3. United Nations. The 2030 Agenda for Sustainable Development's 17 Sustainable Development Goals (SDGs). 2020.

4. Pembe AB, Urassa DP, Darf E, Carlsted A, Olsson P. Qualitative study on maternal referrals in rural Tanzania: decision making and acceptance of referral advice. Afr J Reprod Health. 2008;12:120–31.

5. Awoonor-Williams JK, Bailey PE, Yeji F, Adongo AE, Baffoe P, Williams A, et al. Conducting an audit to improve the facilitation of emergency maternal and newborn referral in northern Ghana. Glob Public Health. 2015;10:1118–33.

6. Ameyaw EK, Njue C, Tran NT, Dawson A. Quality and women's satisfaction with maternal referral practices in sub-Saharan African low and lower-middle income countries: a systematic review. BMC Pregnancy Childbirth. 2020;20:1–16.

7. Ofosu B, Ofori D, Ntumy M, Asah-Opoku K, Boafor T. Assessing the functionality of an emergency obstetric referral system and continuum of care among public healthcare facilities in a low resource setting: an application of process mapping approach. BMC Health Serv Res. 2021;21:1–14.

8. Simba DO, Mbembati NAA, Museru LM, Lema LEK. Referral pattern of patients received at the national referral hospital: challenges in low income countries. East Afr J Public Health. 2008;5:6–9.

9. Proos R, Mathéron H, Vas Nunes J, Falama A, Sery Kamal P, Grobusch MP, et al. Perspectives of health workers on the referral of women with obstetric complications: A qualitative study in rural Sierra Leone. BMJ Open. 2020;10:1–8.

10. Benson AE, Benson MJ, Luke AH. Assessment of maternal referral systems used for a rural Zambian hospital: The development of setting specific protocols for the identification of complications. Afr Health Sci. 2019;19:1536–43.

11. Diba F, Ichsan I, Muhsin M, Marthoenis M, Sofyan H, Andalas M, et al. Healthcare providers' perception of the referral system in maternal care facilities in Aceh, Indonesia: A cross-sectional study. BMJ Open. 2019;9.

12. Afari H, Hirschhorn LR, Michaelis A, Barker P, Sodzi-Tettey S. Quality improvement in emergency obstetric referrals: Qualitative study of provider perspectives in Assin North district, Ghana. BMJ Open. 2014;4.

13. Austin A, Gulema H, Belizan M, Colaci DS, Kendall T, Tebeka M, et al. Barriers to providing quality emergency obstetric care in Addis Ababa, Ethiopia: Healthcare providers' perspectives on training, referrals and supervision, a mixed methods study. BMC Pregnancy Childbirth. 2015;15:1–10.

14. Daniels AA, Abuosi A. Improving emergency obstetric referral systems in low and middle income countries: A qualitative study in a tertiary health facility in Ghana. BMC Health Serv Res. 2020;20:1–10.

15. Give C, Ndima S, Steege R, Ormel H, Mccollum R, Theobald S, et al. Strengthening referral systems in community health programs : a qualitative study in two rural districts of Maputo Province , Mozambique. 2019;:1–11.

16. Pembe AB, Carlstedt A, Urassa DP, Lindmark G, Nyström L, Darj E. Effectiveness of maternal referral system in a rural setting: A case study from Rufiji district, Tanzania. BMC Health Serv Res. 2010;10.

17. Stal KB, Pallangyo P, Elteren M Van, Akker T Van Den, Roosmalen J Van. Women ' s perceptions of the quality of emergency obstetric care in a referral hospital in rural Tanzania.

2015;20:934–40.

18. Mannava P, Durrant K, Fisher J, Chersich M, Luchters S. Attitudes and behaviours of maternal health care providers in interactions with clients: A systematic review. Global Health. 2015;11:1–17.

19. MOHSW T. Policy - National Health Policy. Minist Heal community Dev gender, elderly, Child Tanzania. 2017; October:1–55.

20. NBST. Sensa ya Watu na Makazi ya Mwaka 2022: Matokeo ya Mwanzo. 2022.

21. Clarke V, Braun V. Teaching thematic analysis : Overcoming challenges and developing strategies for effective learning Associate Professor in Sexuality Studies Department of Psychology Faculty of Health and Life Sciences University of the West of England Coldharbour Lane Br. Univ West Engl. 2013;26:120–3.

22. Chodzaza E, Bultemeier K. Service providers' perception of the quality of emergency obsteric care provided and factors indentified which affect the provision of quality care. Malawi Med J. 2010;22:104–11.

23. Yasin C, Geleto A, Berhane Y. Referral linkage among public health facilities in Ethiopia: A qualitative explanatory study of facilitators and barriers for emergency obstetric referral in Addis Ababa city administration. Midwifery. 2019;79.

24. Ankita Kumari, Mitra S, Vani Aditya. Spectrum of obstetric referral and their outcome at a Tertiary Care Center of Eastern Uttar Pradesh: An insight. Asian J Med Sci. 2022;13:123–8.

25. Singh S, Doyle P, Campbell OM, Mathew M, Murthy GVS. Referrals between public sector health institutions for women with obstetric high risk, complications, or emergencies in India - A systematic review. PLoS One. 2016;11:1–23.

26. Bugomola MA. Effectiveness of Maternal Referral System in Tanzania : A Mixed Method Study. 2022;:1–18.

27. Graneheim UH, Lundman B. Qualitative content analysis in nursing research: Concepts,

procedures and measures to achieve trustworthiness. Nurse Educ Today. 2004;24:105–12.

28. Shenton AK. Strategies for Ensuring Trustworthiness in Qualitative Research Projects Strategies for ensuring trustworthiness in qualitative research projects. 2016; February. https://doi.org/10.3233/EFI-2004-22201.

Figure
Click here to access/download;Figure;Fig 1.docx

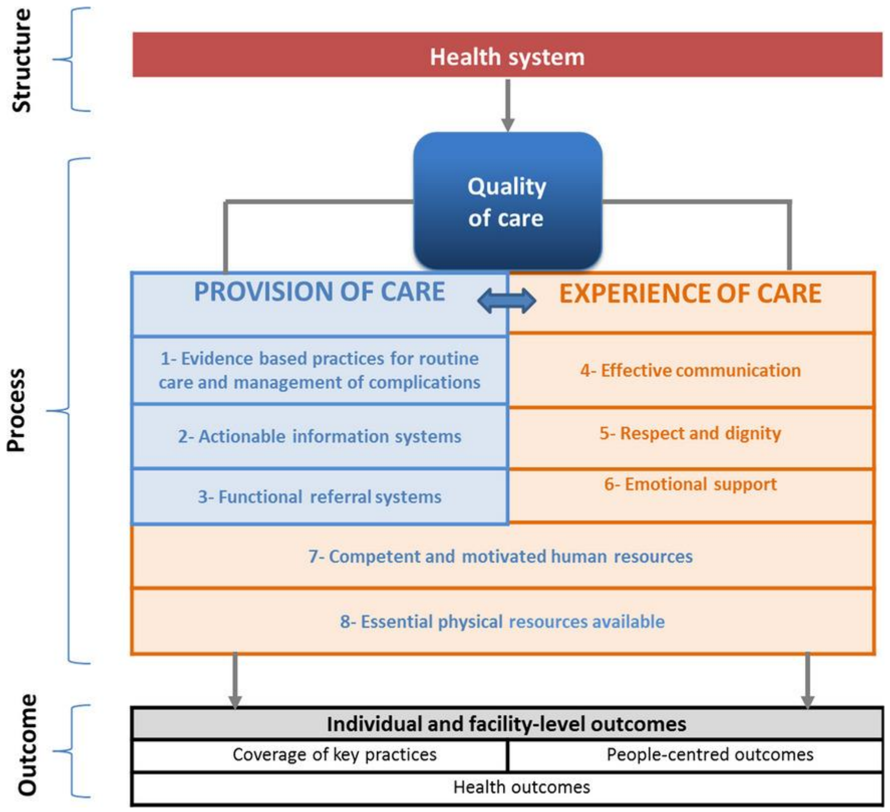

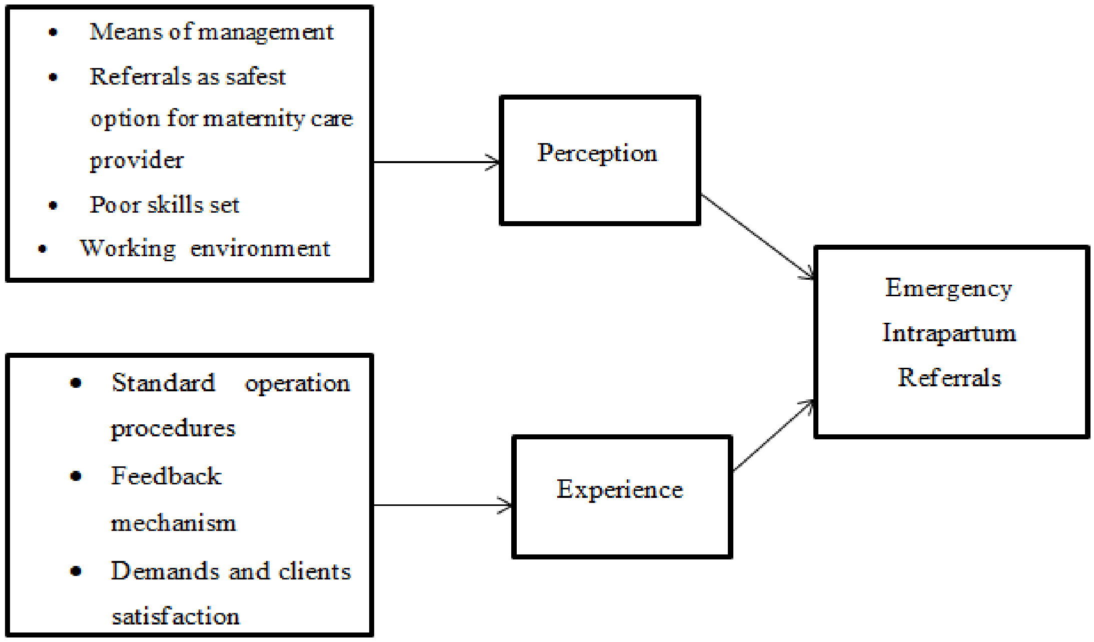
Means of management
Referrals as safest option for maternity care provider
Poor skills set
Working environment
Perception
Emergency Intrapartum Referrals
Standard operation procedures
Feedback mechanism
Demands and clients satisfaction
Experience
