# Supplementary material for: “Are all referrals necessary?” Experiences and perceptions of maternity healthcare providers on emergency intrapartum referrals in Dar es Salaam, Tanzania
Source: PLoS One. 2024 Feb 21;19(2):e0298103. doi: 10.1371/journal.pone.0298103 (PMC10880996; doi:10.1371/journal.pone.0298103)
Supplement: Attachment — Submitted filename: PONE-D-22-34478_R2_All referrals.pdf [file pone.0298103.s004.pdf]

PLOS ONE

“Are all referrals necessary ?” Experiences and perceptions of maternity healthcare providers on emergency intrapartum referrals in Dar es Salaam, Tanzania.

--Manuscript Draft--

**Manuscript Number:**	PONE-D-22-34478R2
**Article Type:**	Research Article
**Full Title:**	“Are all referrals necessary ?” Experiences and perceptions of maternity healthcare providers on emergency intrapartum referrals in Dar es Salaam, Tanzania.
**Short Title:**	Experiences and perceptions of maternity healthcare providers on emergency intrapartum referrals.
**Corresponding Author:**	SHEKHA SELEMANI, MMed<br>Muhimbili University of Health and Allied Sciences School of Medicine<br>DAR ES SALAAM, TANZANIA, UNITED REPUBLIC OF
**Keywords:**	emergency intrapartum referrals; maternity healthcare providers; Tanzania
**Abstract:**	Background: A functional referral system is an important component of quality of care. Continuity of care and reduction of maternal morbidity and mortality rely heavily on effective referral system. Capacity building is expected to improve the referral hierarchy but the situation is different. This study aimed at exploring experiences and perceptions of maternity healthcare providers on emergency intrapartum referrals in Dar es Salaam, Tanzania.<br>Methods: An exploratory qualitative study was conducted in Amana regional referral hospital and Muhimbili national hospital. Maternity healthcare providers were purposively recruited using maximum variation sampling. A semi-structured interview guide was used to conduct eleven in-depth interviews. Thematic analysis approach by Braun and Clarke (2006).<br>Results: Two major themes emerged, namely, referral beyond medical indications and poor communication between referring and receiving facilities hindering care. Referrals, according to maternity healthcare professionals, were seen as a way to minimise blame as well as a clinical management tool to prevent difficulties. It was found that, there was limited understanding on skills set among maternity healthcare providers and some had negative perceptions towards performing their responsibilities.<br>Conclusions: Skills gap among maternity healthcare providers at referring hospitals still existed which influenced the decision making and service provision. There was hostility between referring and receiving hospitals. Strategies such as on-going training and capacity building should be emphasized. The referring hospital should only consider referrals as a last resort.
**Order of Authors:**	SHEKHA SELEMANI, MMed
	MICHAEL O MWAKYUSA, MMed
	SELEMANI BASHIRI, MMed
	MANGI M EZEKIEL, Ph.D
	DORKASI L MWAKAWANGA, MSc
	FADHLUN M ALWY AL-BEITY, Ph.D
	ANDREA B PEMBE, Ph.D
**Opposed Reviewers:**	
**Response to Reviewers:**	Thank you for the comment received.<br>Figure 2 has been referred in the text.<br>I will be grateful to hear from you.
**Additional Information:**	
**Question**	**Response**
**Financial Disclosure**	The author(s) received no specific funding for this work

Powered by Editorial Manager® and ProduXion Manager® from Aries Systems Corporation

Enter a financial disclosure statement that describes the sources of funding for the work included in this submission. Review the submission guidelines for detailed requirements. View published research articles from *PLOS ONE* for specific examples.<br><br>This statement is required for submission and **will appear in the published article** if the submission is accepted. Please make sure it is accurate.<br><br>**Unfunded studies**<br>Enter: *The author(s) received no specific funding for this work.*<br><br>**Funded studies**<br>Enter a statement with the following details:<br>• Initials of the authors who received each award<br>• Grant numbers awarded to each author<br>• The full name of each funder<br>• URL of each funder website<br>• Did the sponsors or funders play any role in the study design, data collection and analysis, decision to publish, or preparation of the manuscript?<br>• **NO** - Include this sentence at the end of your statement: *The funders had no role in study design, data collection and analysis, decision to publish, or preparation of the manuscript.*<br>• **YES** - Specify the role(s) played.<br><br>* typeset	
**Competing Interests**<br><br>Use the instructions below to enter a competing interest statement for this submission. On behalf of all authors, disclose any competing interests that could be perceived to bias this work—acknowledging all financial support and any other relevant financial or non-financial competing interests.<br><br>This statement is required for submission and **will appear in the published article** if	The authors have declared that no competing interests exist

the submission is accepted. Please make sure it is accurate and that any funding sources listed in your Funding Information later in the submission form are also declared in your Financial Disclosure statement.<br><br>View published research articles from *PLOS ONE* for specific examples.<br><br>**NO authors have competing interests**<br><br>Enter: *The authors have declared that no competing interests exist.*<br><br>**Authors with competing interests**<br><br>Enter competing interest details beginning with this statement:<br><br>*I have read the journal's policy and the authors of this manuscript have the following competing interests: [insert competing interests here]*<br><br>* typeset	
**Ethics Statement**<br><br>Enter an ethics statement for this submission. This statement is required if the study involved:<br><br>• Human participants<br>• Human specimens or tissue<br>• Vertebrate animals or cephalopods<br>• Vertebrate embryos or tissues<br>• Field research<br><br>Write "N/A" if the submission does not require an ethics statement.<br><br>General guidance is provided below. Consult the submission guidelines for detailed instructions. **Make sure that all information entered here is included in the Methods section of the manuscript.**	Ethical clearance was obtained from Muhimbili University of Health and Allied Sciences Institutional Review Board (MUHAS-REC-10-2021-867). The permission to conduct the study was obtained from Director of Clinical Services at Amana Regional Referral Hospital and the Director of Clinical Services at Muhimbili National Hospital. Study participants were informed about study goals and objectives and were asked for permission to digitally audio-record. Information provided by the participant was kept confidential at all times and used for the research purpose. All the voices recorded were deleted after transcription and translation of the information. It was guaranteed that information was treated confidentially, anonymised before publication, and that quotes could not be traced back to individuals. We obtained written consent from all participants participated in the study.

**Format for specific study types**<br><br>**Human Subject Research (involving human participants and/or tissue)**<br>• Give the name of the institutional review board or ethics committee that approved the study<br>• Include the approval number and/or a statement indicating approval of this research<br>• Indicate the form of consent obtained (written/oral) or the reason that consent was not obtained (e.g. the data were analyzed anonymously)<br><br>**Animal Research (involving vertebrate animals, embryos or tissues)**<br>• Provide the name of the Institutional Animal Care and Use Committee (IACUC) or other relevant ethics board that reviewed the study protocol, and indicate whether they approved this research or granted a formal waiver of ethical approval<br>• Include an approval number if one was obtained<br>• If the study involved *non-human primates*, add *additional details* about animal welfare and steps taken to ameliorate suffering<br>• If anesthesia, euthanasia, or any kind of animal sacrifice is part of the study, include briefly which substances and/or methods were applied<br><br>**Field Research**<br><br>Include the following details if this study involves the collection of plant, animal, or other materials from a natural setting:<br>• Field permit number<br>• Name of the institution or relevant body that granted permission	
**Data Availability**<br><br>Authors are required to make all data underlying the findings described fully available, without restriction, and from the time of publication. PLOS allows rare exceptions to address legal and ethical concerns. See the PLOS Data Policy and FAQ for detailed information.	No - some restrictions will apply

A Data Availability Statement describing where the data can be found is required at submission. Your answers to this question constitute the Data Availability Statement and **will be published in the article**, if accepted.<br><br>**Important:** Stating 'data available on request from the author' is not sufficient. If your data are only available upon request, select 'No' for the first question and explain your exceptional situation in the text box.<br><br>Do the authors confirm that all data underlying the findings described in their manuscript are fully available without restriction?	
**Describe where the data may be found in full sentences. If you are copying our sample text, replace any instances of XXX with the appropriate details.**<br><br>• If the data are **held or will be held in a public repository**, include URLs, accession numbers or DOIs. If this information will only be available after acceptance, indicate this by ticking the box below. For example: *All XXX files are available from the XXX database (accession number(s) XXX, XXX.*).<br>• If the data are all contained **within the manuscript and/or Supporting Information files**, enter the following: *All relevant data are within the manuscript and its Supporting Information files.*<br>• If neither of these applies but you are able to provide **details of access elsewhere**, with or without limitations, please do so. For example:<br><br>*Data cannot be shared publicly because of [XXX]. Data are available from the XXX Institutional Data Access / Ethics Committee (contact via XXX) for researchers who meet the criteria for access to confidential data.*<br><br>*The data underlying the results presented in the study are available from (include the name of the third party*	Data can not be shared publicly because they contain sensitive participants' informations and participants did not consent to having their interviews shared. Data are available from the Directorate of Research and Publications at the Muhimbili university of health and allied sciences (contact via drp@muhas.ac.tz) for researchers who meet the criteria for access to confidential data.

Powered by Editorial Manager® and ProduXion Manager® from Aries Systems Corporation

and contact information or URL).<br>• This text is appropriate if the data are owned by a third party and authors do not have permission to share the data.<br><br>* typeset	
Additional data availability information:	

MUHIMBILI UNIVERSITY OF HEALTH AND ALLIED SCIENCES

SCHOOL OF MEDICINE

DEPARTMENT OF OBSTETRICS AND GYNECOLOGY

12th, July 2023

Emily Chenette
Editor in Chief
Plos One

Dear Editor:

I am pleased to resubmit our manuscript after addressing well the comment rose from the reviewer, for publication consideration in *Plos One,* titled "***"Are all referrals necessary?"* Experiences and perceptions of maternity healthcare providers on emergency intrapartum referrals in Dar es Salaam, Tanzania**". The paper was co-authored by Michael O. Mwakyusa, Selemani Bashiri, Mangi J. Ezekiel, Dorkasi L. Mwakawanga, Fadhlun M. Alwy Al-beity, and Andrea B. Pembe. Figure 2 has been referred in the text.

This paper describes the experiences and perceptions of maternity healthcare providers on emergency intrapartum referrals. A functional referral system is an important component in reduction of maternal morbidity and mortality. Our study found out that referrals are done beyond medical indications, limited maternity healthcare provider capability at referring facility and there was a limited communication between referring and receiving facilities hindering timely care. Additionally, there was limited understanding on skills set among maternity healthcare providers and some had negative perceptions towards performing their responsibilities.

We confirm that this paper has not been published earlier and is not currently under consideration by another journal. We have read and understood your journal's policies and believe that neither the manuscript nor the study violates any of these. 247 are contained in the abstract. The total number of words in the text is 6412. We possess 2 tables and 2 figures.

There are no conflicts of interest to declare.

Thank you for accepting to review our manuscript. I look forward to hearing from you.

Sincerely,

Shekha Selemani

E-mail: shekha_alinisi@yahoo.com
Corresponding author

Manuscript Click here to access/download;Manuscript;Manuscript.doc

"*Are all referrals necessary*?" Experiences and perceptions of maternity healthcare providers on emergency intrapartum referrals in Dar es Salaam, Tanzania.

Shekha Selemani[1*], Michael O. Mwakyusa[1], Selemani Bashiri[1], Mangi J. Ezekiel[2], Dorkasi L. Mwakawanga[3], Fadhlun M. Alwy Al-beity[1], and Andrea B. Pembe[1]

Authors Affiliations

[1]Department of Obstetrics and Gynecology, Muhimbili University of Health and Allied Sciences, Dar es Salaam, Tanzania.

[2]Department of Behavioral Sciences, Muhimbili University of Health and Allied Sciences, Dar es Salaam, Tanzania.

[3]Department of Community Health Nursing, Muhimbili University of Health and Allied Sciences, Dar es Salaam, Tanzania.

***Correspondence:** Shekha Selemani; Tel: +255782205707

E-mail: shekha_alinisi@yahoo.com

ORCID iD: https://orcid.org/0000-0002-8965-2827

Abstract

Background: A functional referral system is an essential to the quality of care. Continuity of care and maternal morbidity and mortality reduction relies heavily on an effective referral system. Capacity building is expected to improve the referral hierarchy but, the situation is different. This study explored the experiences and perceptions of maternity healthcare providers on emergency intrapartum referrals in Dar es Salaam, Tanzania.

Methods: An exploratory qualitative study was conducted at Amana regional referral hospital and Muhimbili national hospital. Maternity healthcare providers were purposively recruited to ensure maximum variation sampling. We used an in-depth interview guide to conduct eleven in-depth interviews. The thematic analysis approach by Braun and Clarke (2006) guided the data analysis.

Results: Three major themes emerged; namely, referral beyond medical indications, Limited maternity healthcare provider capability at referring facility, and limited communication between referring and receiving facilities. Referrals, according to maternity healthcare professionals, were seen as a way to minimize blame as well as a clinical management tool to prevent difficulties. It was found that there was limited understanding of the skills set among maternity healthcare providers and some had negative perceptions towards performing their responsibilities.

Conclusions: Skills gap among maternity healthcare providers at referring hospitals influenced referral decision-making and service provision. There was hostility between referring and receiving hospitals. Capacity-strengthening strategies such as ongoing skills training and changes in attitudes toward referrals require increased attention. The referring hospital should only consider referrals as a last resort after the other management has been done.

Keywords: Emergency intrapartum referrals, perceptions, experiences, maternity healthcare providers, Tanzania

Introduction

A functional referral system is an important component of the World Health Organization (WHO) quality of care (fig 1) for maternal and newborn health [1, 2]. In 2015, The United Nations launched Sustainable Development Goals target 3.1 which aimed at reducing the global maternal mortality ratio to less than 70 per 100,000 live births by 2030 [3]. An efficient referral system is central and essential for continuity of care, and the reduction of maternal morbidity and mortality [4]. By maximizing the limited health facility capacities, a good and functional referral system increases the efficiency of the health system.

Timely referral to an appropriate health facility for addressing maternity needs is a key indicator of a functional health system [5]. Multi-sectorial collaboration and well-coordinated levels of care, linking communities with essential maternal and newborn care are necessary components of a high-quality referral system [6]. In low and low-middle-income countries, emergency intrapartum referrals service is still a major challenge [7]. In Tanzania, the majority of referrals within the Dar es Salaam region to Muhimbili National Hospital (MNH) have originated from public health facilities. The most common reasons for referral include lack of expertise and necessary equipment; other reasons were lack of drugs and physical space [8].

Referrals are referred to as the safest option for healthcare professionals to prevent devastating consequences such as maternal and fetal morbidity and mortality [9]. However, referrals are often done to avoid complications and deaths within their facility. The woman referred to has been described as “not my case” [9]. A study conducted in rural Zambia minimal consensus on decision-making for specific diseases because no suitable referral strategy was used for each problem [10]. Moreover, excessive requests for delivery care services at health facilities put health providers under pressure, which may influence the number of referrals to tertiary hospitals [11]. A qualitative study done by Afari in Ghana showed that maternity care providers perceived some challenges related to poor quality within their designated skill set which led them to refer patients to tertiary hospitals [12]. A lack of pre-referral communication, the high number of patients at referring hospitals, and the absence of feedback all contribute to an increase in intrapartum referrals and a dysfunctional referral system [13–15].

Studies have been done on the perceptions of women and maternal care providers on the effectiveness and quality of the maternal referral system and obstetric care in referral hospitals.

However, all these studies were conducted in rural settings in Tanzania [4, 16, 17]. Little is known about the perceptions and experiences of maternity care providers on emergency intrapartum referrals in urban settings. Therefore, this study reports the perceptions and experiences of maternity care providers on emergency intrapartum referrals at a regional referral hospital and a tertiary Hospital in Dar es Salaam City, Tanzania.

Fig 1: WHO Quality of Care Framework for Maternal and newborn health

A Conceptual Framework

Fig 2: A conceptual framework illustration

The illustration (fig 2) shows constructs that may influence the perception and experience of maternity care providers on emergency intrapartum referrals. Since we could not find an existing framework for examining the perceptions and experiences of maternity care professionals about emergency intrapartum referrals, we adapted a framework from a relevant study to develop a framework for this study [18]. The framework of this study (fig 2) demonstrates that Standard Operating Procedures availability and usage, patients' demands and satisfaction, patients' attitudes towards maternity care providers, referrals (safest option for healthcare providers), poor skill sets and working environment are among the constructs that may influence perceptions of maternity healthcare providers and resulting into an increment of emergency intrapartum referrals. Competence among maternity healthcare providers and minimal training, working environments such as long working hours, heavy workload, weak supervision and communication with co-workers, and insufficient salaries can contribute to a negative experience among workers, which may also result in a referral of a patient. As a result of a particular experience, maternity healthcare providers at the receiving hospital held a particular view about referrals from a particular facility.

Materials and Methods

Study design and setting

This explorative qualitative study used in-depth interviews to explore maternity healthcare providers' perceptions and experiences on emergency intrapartum referrals in Dar es Salaam

region. The Tanzania health system is a pyramidal structure where a higher hospital level supports a network of lower-level facilities [19]. A district hospital serves as the first referral level for their immediate lower-level facilities health centers and dispensaries. At this level, patients in need of advanced services are transferred to the secondary level hospitals at the regional level and then at the tertiary level which includes zonal, specialized, and National hospitals. The study was done in Dar es Salaam, Tanzania's largest city, with the highest population density of about 5,383,728 [20]. In Tanzania, the healthcare referral system is organized hierarchically: the district hospitals refer patients to regional health facilities that refer patients to the tertiary level health facilities. . Some national guidelines and protocols describe the referral process; however, these are not widely known or followed. This study was done in two referral levels, Amana regional referral hospital, a secondary-level facility, and Muhimbili national hospital, a tertiary-level facility. The two facilities are both located in urban and have similarities and differences in characteristics in capacity, human resources, and infrastructures as seen in **Table 1**.

Table 1. Similarities and differences between the two health facilities in the study

Characteristics	Amana Regional Referral Hospital	Muhimbili National Hospital
Referral level	Secondary level, receiving from district hospitals and large health centers	Tertiary level, receive from three secondary level hospitals, and nearby Pwani regions hospitals such as Bagamoyo, Mkuranga and Kisarawe
Infrastructure	Separate maternity building with antenatal, postnatal, neonatal, and labor wards with six delivery beds<br><br>No separate Intensive Care Unit or High dependence unit<br><br>Dedicated obstetric theatre with two beds within the maternity building	Separate maternity building with antenatal, postnatal, neonatal, and labor wards with six delivery beds<br><br>Has a dedicated Intensive Care Unit and High dependence unit within the maternity building<br>Dedicated obstetric theatre with four beds adjacent to the maternity building
Human resource	Seven medical specialists (obstetric/gynecologist), six registrars, and no residents<br><br>65 midwives of different levels of training (certificate to master's level)<br><br>13 Anesthetist staffs	Twenty-six medical specialists (obstetric/gynecologists and consultants), sixteen registrars, and more than 30 residents who are medical doctors specializing in obstetrics/gynecology.<br><br>159 midwives of different levels of training (diploma to master's level)<br><br>8 Anesthetist staff per shift in obstetric theatre

Daily delivery load	39-45 total deliveries per day	25-35 total deliveries per day
Patient flow	Direct from home and referrals from within the district.	Direct from home and referrals from Primary and secondary level facilities<br><br>Muhimbili National Hospital also has an Intermural Private Practice.

The referral process from Amana to Muhimbili National Hospital

At Amana: Once a referral decision has been made, the specialist on duty or head of the department is consulted to examine the patient and authorize the referral. The nurse supervisor contacts Muhimbili National Hospital, the receiving facility, via telephone. They describe the patient’s diagnosis, condition, reason for referral, and management given. The supervisor organizes the ambulance, and a nurse escorts the woman to the higher-level facility. Private cars are used to transport patients when the ambulance is not available due to a lack of fuel or an absence of a driver. There is a cost associated with renting a private ambulance.

At MNH: the mobile phone stays with the specialist on duty. The specialist carries a dialogue with Amana, provides advice on further interventions, and accepts or redirects the referral to Mloganzila Hospital, which is another branch of the MNH.

When a woman is referred with an intrapartum emergency, she is received at MNH labor ward, where initial management including resuscitations is done. The on-call medical team reassesses and decides on patient disposal, including whether she needs further consultations with senior doctors, interdepartmental consultations, or surgical management.

Study participants and recruitment

A purposive sampling technique was used to recruit maternity healthcare providers of different cadres and levels of training and maternity ward administrators to achieve maximum variation.. The administrators were maternity wards in-charge, the medical officer in charge of the labor ward, and the head of the department from the two facilities. These groups of participants were involved in managing emergency obstetric cases and implementing emergency intrapartum

referral policies. The researcher requested maternity in charge in the two health facilities provide a list of all maternity care providers. The purposive selection was based on three years and above of experience in the department to capture an experience on the referral system, cadre, and their roles in the referral process. Eleven participants including four doctors, five midwives, and two administrators (in charge of maternity wards, head of the department) working at Amana regional referral hospital and MNH participated in the study. The researchers contacted the identified participants in person or by phone to establish rapport and set a day and time for an appointment to participate in interviews.

Data collection

The data were collected in the two hospitals using an in-depth interview guide prepared in English and then translated into Kiswahili language used for conducting interviews. The interview guide contained main questions and probing questions to give a chance to the participant to provide more responses or clarification to what has been said. The first author conducted interviews with the assistance of two research assistants who took notes and audio-recorded the interviews. Research assistants had sociology training and were well conversed in qualitative data collection. Written informed consent was obtained from each participant for taking part in the interview and for the audio recording. Interview procedures were explained and each participant was informed that his/her participation is voluntary and may choose not to answer some of the questions or stop the interview at any time. The in-depth interviews took place in a quiet private room within the respective facility at their convenient time suggested by them. All interviews were conducted in Kiswahili, the native language spoken comfortably by researchers and study participants. All interviews were audio-recorded and lasted between 45 and 60 minutes. The interviews stopped after data saturation was observed when no new information was obtained from the study participants and redundancy had been achieved.

Data processing and analysis

According to Braun and Clarke, the thematic analysis approach involves six steps guiding the data analysis[21]. The analysis was done concurrently with data collection. The analysis began after the completion of the first two individual interviews. Audio recordings of the interviews were transcribed verbatim and then translated into English by the data collection team. The translated transcripts were read through several times to become familiar with the data and to get

an understanding of the participant's accounts. The first author developed the initial coding scheme based on our objective, interview guide, and conceptual understanding of the referral system in Tanzania. To ensure reliability, two researchers coded the first transcript independently and then compared the codes for agreement on the final codes and coding. The coding was done manually. A list of potential and initial codes reflecting the predetermined themes was created through data reduction. Then sub-themes and themes were then generated from the general list of codes created through comparisons. Lastly, themes were reviewed and discussed by all authors, discrepancies were noted, and identified themes were finalized.

Ethical approval and consent to participate

Ethical clearance was obtained from the Muhimbili University of Health and Allied Sciences Institutional Review Board (MUHAS-REC-10-2021-867). The permission to conduct the study was obtained from the Director of Clinical Services at Amana Regional Referral Hospital and the Director of Clinical Services at Muhimbili National Hospital. Study participants were informed about study goals and objectives and were asked for permission to digitally audio-record. Information provided by the participant was kept confidential at all times and used for the research purpose. All the voices recorded were deleted after transcription and translation of the information. It was guaranteed that information was treated confidentially, anonymized before publication, and that quotes could not be traced back to individuals. We obtained written consent from all participants in the study.

Methodological considerations

Trustworthiness in a qualitative study is attained when the findings of such a study are worth believing [27]. Four criteria as explained by Shenton AK, et al. were used to assess the trustworthiness in our study; credibility, dependability, transferability, and conformability [28]. The credibility of findings was ensured through engagement and cross-checking of themes, sub-themes, and findings among research team members. In addition, during the data collection phase, the lead author spent a considerable amount of time interacting with study participants in both hospitals thus getting a better understanding of referral dynamics. The sharing of findings and the description of technical aspects of the qualitative methodologies used to ensure that our results are dependable. Out results are transferable to other contexts since the findings section provides thick descriptions of the procedures, settings, and methods and therefore lend

themselves relevant to other/similar contexts. Our study presents perspectives and experiences on referral from different cadres of staff in the two hospitals and therefore represents a fair range of differing viewpoints (authenticity) on issues around postpartum referrals in Tanzania.

The study achieved rich data through the involvement of maternity Health care providers with experience in the provision of care and referrals in the facilities under study. Furthermore, it provided an in-depth perspective of providers concerning intrapartum referrals thereby identifying gaps and areas for improvement. Despite all these strengths, the study had some limitations. A major limitation is the fact that an assessment of referral guidelines needed to be done to confirm the assertion of the adequacy of the guidelines. Therefore our results should be interpreted in light of this limitation.

Results

We interviewed 11 participants with an age range of 29-56 years. The average age of the participants was 40 years, where 45% were female and 55% were male. Among them, five were midwives, two registrars, three obstetrics and gynecologists, and one administrator. All of them had a working experience of at least three years.

Three main themes emerged from the analysis process each with 2 to 5 sub-themes: (1) Referral beyond medical indications (2) Limited maternity healthcare provider capability at referring facility (3) Limited communication between referring and receiving facilities. **See Table 2.**

Table 2: Themes and Sub-themes

**Themes**	**Sub-Themes**
Referral beyond medical indications	- Referral process<br>- Referrals due to a mismatch between delivery load and available facilities<br>- Referrals due to challenges in

	infrastructures<br>- Referrals to avoid blame<br>- Lack of motivation
Limited maternity healthcare provider capability at referring facility	- Lack of basic knowledge and skills in practices among maternity healthcare providers (MHP)<br>- The irresponsibility of MHPs at referring facility
Limited communication between referring and receiving facilities	- Harsh treatment to an escort nurse<br>- Lack of constructive feedback to the referring facilities

Theme 1. Referral beyond medical indications

This theme illustrates about experiences of participants on how the decision for referral is being made and other reasons for referrals. Five subthemes are described under this theme; the process of referral, referrals due to a mismatch between delivery load and available facilities, referrals due to challenges in infrastructures, referral to avoid blames, and lack of motivation.

The process of referral

The majority of referrals made were emergency intrapartum referrals. Before referral, standard operating procedures were not always followed. Participant stated that patients were referred without the required pre-referral management or appropriate documentation on the referral form. There was an increase in the number of referrals during the night compared to the day, and it was reported that poor supervision of junior doctors during the night affected decision- making and referrals at a late stage. A participant from receiving hospital was quoted saying,

"… A patient brought with an emergency condition having 18-20 gauges or resuscitated with 5 liters of fluid but came with no catheter and the patient is bleeding, you consider this as being lazy or they don't know their work…" (Administrator, receiving hospital)

It was responded with a participant from referring hospital was quoted saying,

"… sometimes we receive many patients at once who need to be referred, so following SOPs before referral might be a difficulty, otherwise, referrals must be issued out by the doctor upon agreement, and a referral form will be signed" (Overall in-charge, referring hospital).

Referrals due to a mismatch between delivery load and available facilities

It was believed by participants that the overwhelming number of patients at referring facilities influenced the decision to provide referrals. The hospital's infrastructure, including its operating rooms, neonatal and maternal intensive care units, and blood and transfusion product availability, precludes it from functioning as a referral hospital, which discourages it from providing referrals as a means of receiving assistance. A participant reported,

"… Right now, we have a big number of patients that we receive who are real emergencies but the environment itself in this hospital is not suitable to receive all of them…" (Specialist, referring hospital)

Another participant was quoted saying,

"… a patient referred from Mbagala Rangi Tatu hospital to Temeke hospital and straight was referred to Muhimbili hospital, that shows there was high a number of patients at Temeke that they couldn't manage that patient from Mbagala…" (Midwife, receiving hospital).

Referrals due to challenges in infrastructure

There was an unavailability of infrastructure such as intensive care units, high dependency units, enough operating rooms, the blood supply for transfusion, and an ambulance. When more than one emergency arises, then referral had to be issued. In addition, because of the availability of one ambulance, then they preferred to call for a referral at Upanga (a nearby national hospital

branch) compared to Mloganzila (a distant national hospital branch) because of the distance factor. To drive this point home, a participant quoted saying,

> *"When I was on duty yesterday, there was an urgent need for treatment for both an ectopic patient and a PPH patient, so I was compelled to take the PPH patient into the operating room while sending the Ectopic patient to a different hospital because there was no space to serve both patients" (Registrar, referring hospital).*

Another participant from receiving hospital said,

> *"A referral made early for a woman with fetal distress or two prior scars in labor, but the patient is brought 6 or 8 hours later, only to find out about the difficulties in finding an ambulance or an escort nurse who must cycle between more than two hospitals." (Midwife, receiving hospital)*

Referrals to avoid blame

When a patient or patient's relative is a member of the medical profession or has government influence, they frequently demand a referral for no valid reason. The majority of these obstacles are faced by hospital administrators who must endure the burden of receiving phone calls from patients demanding a referral.

> *"…But when a relative wants or is in favor of getting a referral to her relative and then you decide to retain a patient, when things go wrong, you can take the blame" (Administrator, referring hospital).*

Another participant added by saying, *"…we sometimes give a referral to the patient whom we could manage here, only to avoid delays and blames…" (Registrar, referring hospital.*

Lack of motivation

It was reported when providers did not receive their allowance payments on time, their motivation decreased, resulting in sub-optimal performance. Some doctors had a decreased desire to manage patients who visited a referring hospital during their shift. So that he/she could rest, he/she would choose to see some of the patients and refer other patients. Either participant reported that other doctors routinely disregard SOPs and refer patients without a genuine reason. A participant mentioned,

> *"We receive some referrals without an informing call and no convincing reason of referral, some doctors will just refer the patient knowing when the patient reached receiving hospital will never be rejected and taken back to our hospital" (registrar, receiving hospital).*

Theme 2: Limited maternity healthcare provider capability at referring facility

This theme illustrates the perception of MHPs' ability in the referring hospital. Two subthemes were identified: lack of basic knowledge and skills MHPs and irresponsibility in treating patients.

Lack of basic knowledge and skills in practices among MHPs at referring facility

Inadequate knowledge and skills among maternal care providers were found to exist, and some were incompetent in performing their responsibilities. Participants reported that when junior doctors failed to consult their seniors, ended up making wrong decisions that consequently caused delays in the provision of management to the patient.

> *"... Most of the time there is a gap, you find an intern doctor can't decide and still does not consult. At the end, decisions for referral are made very late" (Specialist, referring hospital)*

A participant from receiving hospital was quoted saying, *"...as I was working in the labor ward, there was a patient referred with a diagnosis of a previous scar with impending rupture, but after our investigation she was found to have umbilical hernia..." (Midwife, receiving hospital)*

The irresponsibility of MHPs at referring facility

Participants at the receiving hospital perceived the MHPs at the referring hospital as irresponsible since they sometimes refer patients unnecessarily. They further expressed that, sometimes upon phone calls they mention to refer two or three patients but they end up bringing four to five patients because they want to lessen the workload. One participant said:

> *" ... maybe they receive many patients, and they fail to attend all of them. You find there is no genuine reason to refer them. They call for referral of two patients and they bring 4 of them..." (Overall in-charge, receiving hospital).*

Theme 3. Limited communication between referring and receiving facilities

This theme illustrates the communication challenges among MHPs from the referring and receiving hospitals. These include harsh treatment of the MHPs by the referring hospital and lacking constructive feedback from the receiving hospital to the referring hospital.

Harsh treatment to an escort nurse

Participants state that an escort nurse who arrived with the patient at receiving hospital sometimes was less informed about the condition of the patient and the referral form was not filled properly. The predicament led the maternity staff at the receiving hospital to act hostilely against the escort nurses by presuming they would not do their duties as requested. A participant reported,

> *" ... I was called to escort the patient while I was in another ward, it was hard to know all the patient's information, especially if there were many patients. When you reached there, the staff acted haughtily and asked questions as if we didn't want to fulfill our obligations, even though that is not the case…" (Midwife, referring hospital).*

Lack of constructive feedback to the referring facilities

Participants reported that feedback from the receiving hospital was only sometimes given. Sometimes it was given as a report which tends to end up at the administration level and not reach the Maternity healthcare providers. For mismanaged patients who ended up in maternal death, feedback was given through phone calls.

> *"I was thinking there should be a procedure of sending feedback to the maternal care providers on the patients they referred and their outcome, we do write weekly reports, but it ended up at the managerial level and not reaching to the providers" (Midwife, receiving hospital)*

When a woman with an intrapartum emergency was referred to a tertiary hospital, she was received at the labor ward, where initial management, including resuscitations, was initiated. The on-call medical team reassesses and decides on patient disposal: whether she needs further consultations with senior doctors as inter-departmental consultations or surgical management within the maternity department. Unfortunately, no feedback information was sent back to the referring hospital regarding the progress of the management of the patient. A participant from referring hospital had this to say, "*…They hardly ever bring us feedback or let us, the providers, receive it. Feedback rarely reaches the service providers …" (Registrar, referring hospital)*

Discussion

This study explored the perceptions and experiences of maternity care providers regarding intrapartum emergency referrals in a regional referral hospital and Muhimbili National Hospital in Dar es Salaam, Tanzania. In response to our in-depth interviews, we found out that referrals beyond medical indications, such as referring a patient to avoid blame and deaths occurring at a particular health facility, limited competencies and irresponsibility of maternity healthcare providers, and limited communication between a referring and receiving health facility such as lacking constructive feedback and use of harsh language to escort health personnel were found to be the main findings.

Our study found that decision-making for the provision of referrals were influenced by high patient demand for quality service delivery. When a patient was health personnel or had an influence from the maternal care providers or from the government to obtain a referral without

an apparent reason[redacted] Since everyone wishes to receive the best care and the majority of patients believe the national hospital provides the best care, they demand referrals even without a genuine reason. Studies conducted in Indonesia and Tanzania [9, 11] found that providers perceived patients to be overly demanding during the referral process with the pressure they exerted on the delivery of services. In addition, the patient's perceptions of the hospital's services place them under duress and have an effect on the referrals made. The relationship between the patient and provider is crucial to the delivery of quality services.

Intrapartum is a very crucial period that requires close care and monitoring to ensure the safety of both the mother and the baby. To meet patient demands and prevent adverse outcomes, maternal care providers often refer patients who need an apparent reason to avoid blame and delays and sometimes make referrals without communication with the receiving facility beforehand[11]. Similar findings on the referral to avoid delays and blames were found in a Sierra Leone study [12], which showed that some referrals were made to avoid complications or maternal death at their facility. Maternal care providers may refer a patient to avoid liability in the event of adverse outcomes or after receiving negative feedback. This could be explained by fear of damaging their reputation as MHPs and hospitals if a negative outcome happened.

The unfriendly working environment which lacked intensive care units and high dependency units for adults and neonates, a sufficient blood supply for transfusion, a sufficient number of operating rooms, a sufficient number of staffs, and timely payment of allowances lowered their motivation at work. Similar studies report staff shortage as a significant challenge influencing referrals [11, 16, 22]. Studies done in Ghana, Ethiopia and Indonesia [11, 16, 23] revealed similar findings regarding the need for more sufficient infrastructure and staff, which contributing to patient referral. The unconducive working environment contributed significantly to stress, frustrations, fatigue, low job satisfaction, and loss of morale. When providers are not paid their allowances on time, their morale to work at a high level is diminished. This could be because the government has upgraded these hospitals to referral hospitals, where the numbers of patients and workload have increased, but the working environment and staffing levels remained the same. It brought the challenge of increasing the number of referrals to the tertiary hospitals. This reduces the quality of services provided at tertiary hospitals because of needing more support.

In our study, some providers needed to gain skills in managing patients. These providers, who may be newly employed, interns, or those who were resistant to learning, caused delays in providing proper management or incorrect referral decisions. This could be explained by the need for more lack of adequate supervision from their seniors, who were not available at night. Similar studies reported that inadequate training, which leads to a lack of skills and inadequate compensation facilitates maternal care providers to refer patients to another facility [18]. Other studies [12, 24] showed that low quality within their designated skill set made influenced providers in referring women with intrapartum emergencies. Some colleagues were incompetent in making patients' diagnosis because he/she had little work experience compared to the senior provider; consequently, they tend to refer more patients due to a lack of skills and confusion regarding clinical referral criteria [23, 25]. The similarity may be due to the lack of motivation of the seniors for not being paid their allowances timely, so they opt to work during the day time and use other time for part time jobs to supplement their income.

Feedback provides an opportunity for providers to learn where they did wrong or the proper way to handle various cases, thereby improving the quality of services. According to our study, feedback was only sometimes provided, and there was no formal way of providing feedback. This could be a weakness on the part of the administration, wherein responsible personnel failed to perform their duties or raise a challenge if they had any, regarding the delivery of feedback. Studies highlighted the lack of regular feedback to referring facilities and inconsistent use of feedback forms, instead, only complaints about what needed to be appropriately done or expected to be done were reported [14, 26]. Other studies with comparable findings on referral feedback demonstrated that referring facilities were not always provided with feedback, and that feedback was sometimes given to the women or other close family members [6]. The lack of standard national referral guidelines had made no uniformity in delivering services, especially in giving feedback which is a barrier to a functional referral system [15].

Conclusion

Our study found that there was referral hostility between referring and receiving hospitals. The skills gap among maternal care providers at referring hospitals still existed which influencing the decision- making and provision of quality services. Referral should be regarded as the last option

after the patient has been managed to the best of knowledge at referring hospital, and pre-referral management should be provided before referral. Strategies to strengthen communication between the hospitals are warranted. These include ongoing training should be emphasized. Standard referral guidelines should be developed so that each hospital would know their roles and expectations. Further studies on the experiences of women and their relatives on referral services should be done, to get a clear picture of where we are lacking and what needs to be done.

Acknowledgments

The authors acknowledge the support received from all the participants in this study. We also thank Amana Regional Referral Hospital and Muhimbili National Hospital for granting access to use their facilities in this study.

Authors' contributions

SS conceptualized the study, conducted data collection, and analysis, and wrote the original draft. ABP and MME aided the conceptualization and analysis and supervised the writing. DLM and FAA participated in the analysis, writing, and reviewing the manuscript. MOM and SB edited and reviewed the manuscript. All authors reviewed and approved the final manuscript.

Conflict of interest

The authors declare that they have no conflicting interests.

Data availability

Data cannot be shared publicly because they contain sensitive participants' information and participants did not consent to having their interviews shared. Data are available from the Directorate of Research and Publications at the Muhimbili University of Health and Allied Sciences (contact via drp@muhas.ac.tz) for researchers who meet the criteria for access to confidential data.

Funding

The author (s) received no specific funding for this work.

References

1. WHO. Quality, Equity, Dignity: A Network for Improving Quality of Care for Maternal, Newborn and Child Health. 2017; February:14–6.

2. TDHS. Tanzania Demographic and Health Survey Indicator Survey (TDHS-MIS) 2015-2016. Dar es Salaam, Tanzania, Rockville, Maryland, USA MoHCDGEC, MoH, NBS, OCGS, ICF. 2016;1:1–630.

3. United Nations. The 2030 Agenda for Sustainable Development's 17 Sustainable Development Goals (SDGs). 2020.

4. Pembe AB, Urassa DP, Darf E, Carlsted A, Olsson P. Qualitative study on maternal referrals in rural Tanzania: decision making and acceptance of referral advice. Afr J Reprod Health. 2008;12:120–31.

5. Awoonor-Williams JK, Bailey PE, Yeji F, Adongo AE, Baffoe P, Williams A, et al. Conducting an audit to improve the facilitation of emergency maternal and newborn referrals in northern Ghana. Glob Public Health. 2015;10:1118–33.

6. Ameyaw EK, Njue C, Tran NT, Dawson A. Quality and women's satisfaction with maternal referral practices in sub-Saharan African low and lower-middle income countries: a systematic review. BMC Pregnancy Childbirth. 2020;20:1–16.

7. Ofosu B, Ofori D, Ntumy M, Asah-Opoku K, Boafor T. Assessing the functionality of an

emergency obstetric referral system and continuum of care among public healthcare facilities in a low resource setting: an application of process mapping approach. BMC Health Serv Res. 2021;21:1–14.

8. Simba DO, Mbembati NAA, Museru LM, Lema LEK. Referral pattern of patients received at the national referral hospital: challenges in low- income countries. East Afr J Public Health. 2008;5:6–9.

9. Proos R, Mathéron H, Vas Nunes J, Falama A, Sery Kamal P, Grobusch MP, et al. Perspectives of health workers on the referral of women with obstetric complications: A qualitative study in rural Sierra Leone. BMJ Open. 2020;10:1–8.

10. Benson AE, Benson MJ, Luke AH. Assessment of maternal referral systems used for a rural Zambian hospital: The development of setting specific protocols for the identification of complications. Afr Health Sci. 2019;19:1536–43.

11. Diba F, Ichsan I, Muhsin M, Marthoenis M, Sofyan H, Andalas M, et al. Healthcare providers' perception of the referral system in maternal care facilities in Aceh, Indonesia: A cross-sectional study. BMJ Open. 2019;9.

12. Afari H, Hirschhorn LR, Michaelis A, Barker P, Sodzi-Tettey S. Quality improvement in emergency obstetric referrals: Qualitative study of provider perspectives in Assin North district, Ghana. BMJ Open. 2014;4.

13. Austin A, Gulema H, Belizan M, Colaci DS, Kendall T, Tebeka M, et al. Barriers to providing quality emergency obstetric care in Addis Ababa, Ethiopia: Healthcare providers' perspectives on training, referrals and supervision, a mixed methods study. BMC Pregnancy Childbirth. 2015;15:1–10.

14. Daniels AA, Abuosi A. Improving emergency obstetric referral systems in low and middle-income countries: A qualitative study in a tertiary health facility in Ghana. BMC Health Serv Res. 2020;20:1–10.

15. Give C, Ndima S, Steege R, Ormel H, Mccollum R, Theobald S, et al. Strengthening referral systems in community health programs : a qualitative study in two rural districts of Maputo

Province, Mozambique. 2019;:1–11.

16. Pembe AB, Carlstedt A, Urassa DP, Lindmark G, Nyström L, Darj E. Effectiveness of maternal referral system in a rural setting: A case study from Rufiji district, Tanzania. BMC Health Serv Res. 2010;10.

17. Stal KB, Pallangyo P, Elteren M Van, Akker T Van Den, Roosmalen J Van. Women s' perceptions of the quality of emergency obstetric care in a referral hospital in rural Tanzania. 2015;20:934–40.

18. Mannava P, Durrant K, Fisher J, Chersich M, Luchters S. Attitudes and behaviors of maternal health care providers in interactions with clients: A systematic review. Global Health. 2015;11:1–17.

19. MOHSW T. Policy - National Health Policy. Minist Heal community Dev gender, elderly, Child Tanzania. 2017; October:1–55.

20. NBST. Sensa ya Watu na Makazi ya Mwaka 2022: Matokeo ya Mwanzo. 2022.

21. Clarke V, Braun V. Teaching thematic analysis : Overcoming challenges and developing strategies for effective learning Associate Professor in Sexuality Studies Department of Psychology Faculty of Health and Life Sciences University of the West of England Coldharbour Lane Br. Univ West Engl. 2013;26:120–3.

22. Chodzaza E, Bultemeier K. Service providers' perception of the quality of emergency obstetric care provided and factors identified which affect the provision of quality care. Malawi Med J. 2010;22:104–11.

23. Yasin C, Geleto A, Berhane Y. Referral linkage among public health facilities in Ethiopia: A qualitative explanatory study of facilitators and barriers for emergency obstetric referral in Addis Ababa city administration. Midwifery. 2019;79.

24. Ankita Kumari, Mitra S, Vani Aditya. The spectrum of obstetric referral and their outcome at a Tertiary Care Center of Eastern Uttar Pradesh: An insight. Asian J Med Sci. 2022;13:123–8.

25. Singh S, Doyle P, Campbell OM, Mathew M, Murthy GVS. Referrals between public sector health institutions for women with obstetric high risk, complications, or emergencies in India - A systematic review. PLoS One. 2016;11:1–23.

26. Bugomola MA. Effectiveness of Maternal Referral System in Tanzania : A Mixed Method Study. 2022;:1–18.

27. Graneheim UH, Lundman B. Qualitative content analysis in nursing research: Concepts, procedures and measures to achieve trustworthiness. Nurse Educ Today. 2004;24:105–12.

28. Shenton AK. Strategies for Ensuring Trustworthiness in Qualitative Research Projects Strategies for ensuring trustworthiness in qualitative research projects. 2016; February. https://doi.org/10.3233/EFI-2004-22201.

 Click here to access/download;Figure;Fig 1.docx

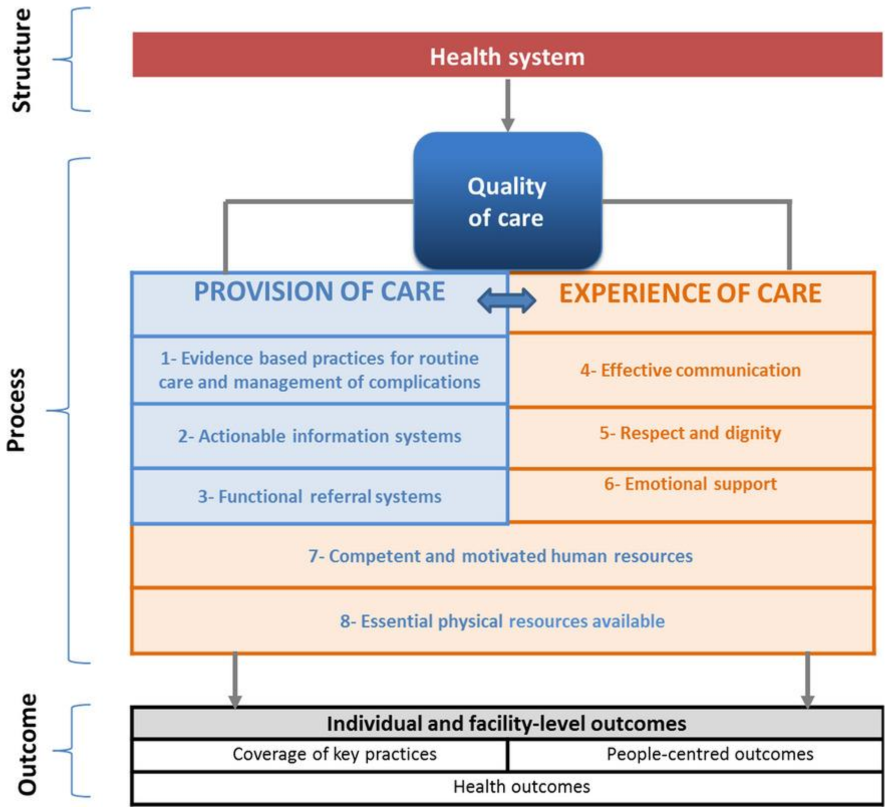

Click here to access/download;Figure;Fig 2.docx

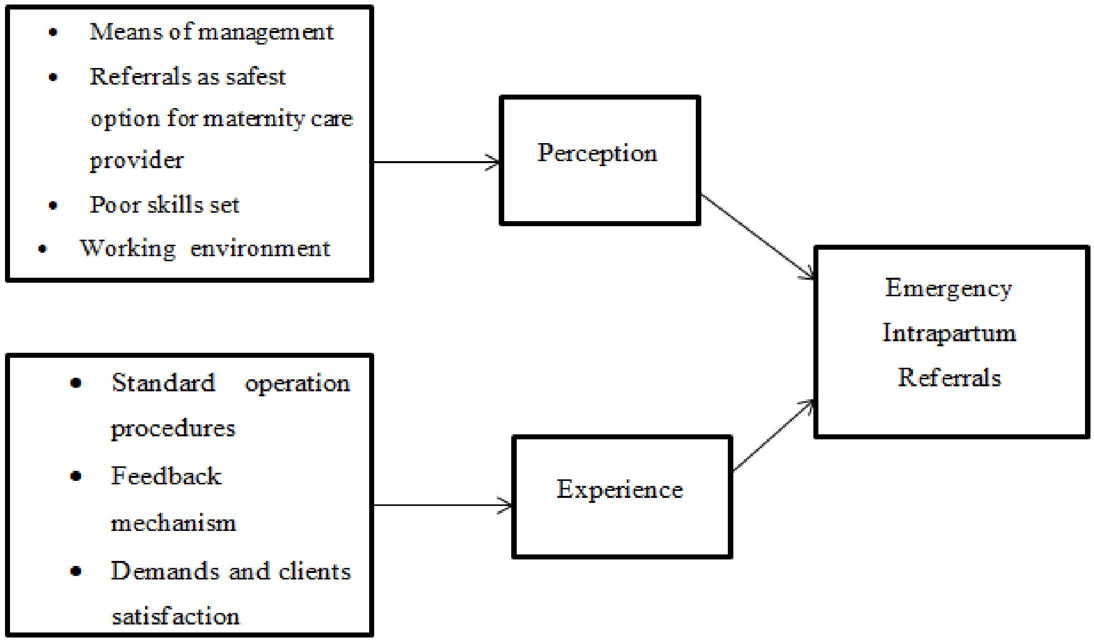

"*Are all referrals necessary*?" Experiences and perceptions of maternity healthcare providers on emergency intrapartum referrals in Dar es Salaam, Tanzania.

Shekha Selemani[1*], Michael O. Mwakyusa[1], Selemani Bashiri[1], Mangi J. Ezekiel[2], Dorkasi L. Mwakawanga[3], Fadhlun M. Alwy Al-beity[1], and Andrea B. Pembe[1]

Authors Affiliations

[1]Department of Obstetrics and Gynecology, Muhimbili University of Health and Allied Sciences, Dar es Salaam, Tanzania.

[2]Department of Behavioral Sciences, Muhimbili University of Health and Allied Sciences, Dar es Salaam, Tanzania.

[3]Department of Community Health Nursing, Muhimbili University of Health and Allied Sciences, Dar es Salaam, Tanzania.

***Correspondence:** Shekha Selemani; Tel: +255782205707

E-mail: shekha_alinisi@yahoo.com

ORCID iD: https://orcid.org/0000-0002-8965-2827

Abstract

Background: A functional referral system is an essential to the quality of care. Continuity of care and maternal morbidity and mortality reduction relies heavily on an effective referral system. Capacity building is expected to improve the referral hierarchy but, the situation is different. This study explored the experiences and perceptions of maternity healthcare providers on emergency intrapartum referrals in Dar es Salaam, Tanzania.

Methods: An exploratory qualitative study was conducted at Amana regional referral hospital and Muhimbili national hospital. Maternity healthcare providers were purposively recruited to ensure maximum variation sampling. We used an in-depth interview guide to conduct eleven in-depth interviews. The thematic analysis approach by Braun and Clarke (2006) guided the data analysis.

Results: Three major themes emerged; namely, referral beyond medical indications, Limited maternity healthcare provider capability at referring facility, and limited communication between referring and receiving facilities. Referrals, according to maternity healthcare professionals, were seen as a way to minimize blame as well as a clinical management tool to prevent difficulties. It was found that there was limited understanding of the skills set among maternity healthcare providers and some had negative perceptions towards performing their responsibilities.

Conclusions: Skills gap among maternity healthcare providers at referring hospitals influenced referral decision-making and service provision. There was hostility between referring and receiving hospitals. Capacity-strengthening strategies such as ongoing skills training and changes in attitudes toward referrals require increased attention. The referring hospital should only consider referrals as a last resort after the other management has been done.

Keywords: Emergency intrapartum referrals, perceptions, experiences, maternity healthcare providers, Tanzania

Introduction

A functional referral system is an important component of the World Health Organization (WHO) quality of care (fig 1) for maternal and newborn health [1, 2]. In 2015, The United Nations launched Sustainable Development Goals target 3.1 which aimed at reducing the global maternal mortality ratio to less than 70 per 100,000 live births by 2030 [3]. An efficient referral system is central and essential for continuity of care, and the reduction of maternal morbidity and mortality [4]. By maximizing the limited health facility capacities, a good and functional referral system increases the efficiency of the health system.

Timely referral to an appropriate health facility for addressing maternity needs is a key indicator of a functional health system [5]. Multi-sectorial collaboration and well-coordinated levels of care, linking communities with essential maternal and newborn care are necessary components of a high-quality referral system [6]. In low and low-middle-income countries, emergency intrapartum referrals service is still a major challenge [7]. In Tanzania, the majority of referrals within the Dar es Salaam region to Muhimbili National Hospital (MNH) have originated from public health facilities. The most common reasons for referral include lack of expertise and necessary equipment; other reasons were lack of drugs and physical space [8].

Referrals are referred to as the safest option for healthcare professionals to prevent devastating consequences such as maternal and fetal morbidity and mortality [9]. However, referrals are often done to avoid complications and deaths within their facility. The woman referred to has been described as “not my case” [9]. A study conducted in rural Zambia minimal consensus on decision-making for specific diseases because no suitable referral strategy was used for each problem [10]. Moreover, excessive requests for delivery care services at health facilities put health providers under pressure, which may influence the number of referrals to tertiary hospitals [11]. A qualitative study done by Afari in Ghana showed that maternity care providers perceived some challenges related to poor quality within their designated skill set which led them to refer patients to tertiary hospitals [12]. A lack of pre-referral communication, the high number of patients at referring hospitals, and the absence of feedback all contribute to an increase in intrapartum referrals and a dysfunctional referral system [13–15].

Studies have been done on the perceptions of women and maternal care providers on the effectiveness and quality of the maternal referral system and obstetric care in referral hospitals.

However, all these studies were conducted in rural settings in Tanzania [4, 16, 17]. Little is known about the perceptions and experiences of maternity care providers on emergency intrapartum referrals in urban settings. Therefore, this study reports the perceptions and experiences of maternity care providers on emergency intrapartum referrals at a regional referral hospital and a tertiary Hospital in Dar es Salaam City, Tanzania.

Fig 1: WHO Quality of Care Framework for Maternal and newborn health

A Conceptual Framework

Fig 2: A conceptual framework illustration

The illustration (fig 2) shows constructs that may influence the perception and experience of maternity care providers on emergency intrapartum referrals. Since we could not find an existing framework for examining the perceptions and experiences of maternity care professionals about emergency intrapartum referrals, we adapted a framework from a relevant study to develop a framework for this study [18]. The framework of this study (fig 2) demonstrates that Standard Operating Procedures availability and usage, patients' demands and satisfaction, patients' attitudes towards maternity care providers, referrals (safest option for healthcare providers), poor skill sets and working environment are among the constructs that may influence perceptions of maternity healthcare providers and resulting into an increment of emergency intrapartum referrals. Competence among maternity healthcare providers and minimal training, working environments such as long working hours, heavy workload, weak supervision and communication with co-workers, and insufficient salaries can contribute to a negative experience among workers, which may also result in a referral of a patient. As a result of a particular experience, maternity healthcare providers at the receiving hospital held a particular view about referrals from a particular facility.

Materials and Methods

Study design and setting

This explorative qualitative study used in-depth interviews to explore maternity healthcare providers' perceptions and experiences on emergency intrapartum referrals in Dar es Salaam

region. The Tanzania health system is a pyramidal structure where a higher hospital level supports a network of lower-level facilities [19]. A district hospital serves as the first referral level for their immediate lower-level facilities health centers and dispensaries. At this level, patients in need of advanced services are transferred to the secondary level hospitals at the regional level and then at the tertiary level which includes zonal, specialized, and National hospitals. The study was done in Dar es Salaam, Tanzania's largest city, with the highest population density of about 5,383,728 [20]. In Tanzania, the healthcare referral system is organized hierarchically: the district hospitals refer patients to regional health facilities that refer patients to the tertiary level health facilities. . Some national guidelines and protocols describe the referral ~~process,~~process; however, these are not widely known or followed. This study was done in two referral levels, Amana regional referral hospital, a secondary-level facility, and Muhimbili national hospital, a tertiary-level facility. The two facilities are both located in urban and have similarities and differences in characteristics in capacity, human resources, and infrastructures as seen in **Table 1**.

Table 1. Similarities and differences between the two health facilities in the study

Characteristics	Amana Regional Referral Hospital	Muhimbili National Hospital
Referral level	Secondary level, receiving from district hospitals and large health centers	Tertiary level, receive from three secondary level hospitals, and nearby Pwani regions hospitals such as Bagamoyo, Mkuranga and Kisarawe
Infrastructure	Separate maternity building with antenatal, postnatal, neonatal, and labor wards with six delivery beds<br><br>No separate Intensive Care Unit or High dependence unit<br><br>Dedicated obstetric theatre with two beds within the maternity building	Separate maternity building with antenatal, postnatal, neonatal, and labor wards with six delivery beds<br><br>Has a dedicated Intensive Care Unit and High dependence unit within the maternity building<br>Dedicated obstetric theatre with four beds adjacent to the maternity building
Human resource	Seven medical specialists (obstetric/gynecologist), six registrars, and no residents<br><br>65 midwives of different levels of training (certificate to master's level)<br><br>13 Anesthetist staffs	Twenty-six medical specialists (obstetric/gynecologists and consultants), sixteen registrars, and more than 30 residents who are medical doctors specializing in obstetrics/gynecology.<br><br>159 midwives of different levels of training (diploma to master's level)<br><br>8 Anesthetist staff per shift in obstetric theatre

Daily delivery load	39-45 total deliveries per day	25-35 total deliveries per day
Patient flow	Direct from home and referrals from within the district.	Direct from home and referrals from Primary and secondary level facilities<br>Muhimbili National Hospital also has an Intermural Private Practice.

The referral process from Amana to Muhimbili National Hospital

At Amana: Once a referral decision has been made, the specialist on duty or head of the department is consulted to examine the patient and authorize the referral. The nurse supervisor contacts Muhimbili National Hospital, the receiving facility, via telephone. They describe the patient's diagnosis, condition, reason for referral, and management given. The supervisor organizes the ambulance, and a nurse escorts the woman to the higher-level facility. Private cars are used to transport patients when the ambulance is not available due to a lack of fuel or an absence of a driver. There is a cost associated with renting a private ambulance.

At MNH: the mobile phone stays with the specialist on duty. The specialist carries a dialogue with Amana, provides advice on further interventions, and accepts or redirects the referral to Mloganzila Hospital, which is another branch of the MNH.

When a woman is referred with an intrapartum emergency, she is received at MNH labor ward, where initial management including resuscitations is done. The on-call medical team reassesses and decides on patient disposal, including whether she needs further consultations with senior doctors, interdepartmental consultations, or surgical management.

Study participants and recruitment

A purposive sampling technique was used to recruit maternity healthcare providers of different cadres and levels of training and maternity ward administrators to achieve maximum variation.. The administrators were maternity wards in-charge, the medical officer in charge of the labor ward, and the head of the department from the two facilities. These groups of participants were involved in managing emergency obstetric cases and implementing emergency intrapartum

referral policies. The researcher requested maternity in charge in the two health facilities provide a list of all maternity care providers. The purposive selection was based on three years and above of experience in the department to capture an experience on the referral system, cadre, and their roles in the referral process. Eleven participants including four doctors, five midwives, and two administrators (~~incharge~~in charge of maternity wards, head of the department) working at Amana regional referral hospital and MNH participated in the study. The researchers contacted the identified participants in person or by phone to establish rapport and set a day and time for an appointment to participate in interviews.

Data collection

The data were collected in the two hospitals using an in-depth interview guide prepared in English and then translated into Kiswahili language used for conducting interviews. The interview guide contained main questions and probing questions to give a chance to the participant to provide more responses or clarification to what has been said. The first author conducted interviews with the assistance of two research assistants who took notes and audio-recorded the interviews. Research assistants had sociology training and were well conversed in qualitative data collection. Written informed consent was obtained from each participant for taking part in the interview and for the audio recording. Interview procedures were explained and each participant was informed that his/her participation is voluntary and may choose not to answer some of the questions or stop the interview at any time. The in-depth interviews took place in a quiet private room within the respective facility at their convenient time suggested by them. All interviews were conducted in Kiswahili, the native language spoken comfortably by researchers and study participants. All interviews were audio-recorded and lasted between 45 and 60 minutes. The interviews stopped after data saturation was observed when no new information was obtained from the study participants and redundancy had been achieved.

Data processing and analysis

According to Braun and Clarke, the thematic analysis approach involves six steps guiding the data analysis[21]. The analysis was done concurrently with data collection. The analysis began after the completion of the first two individual interviews. Audio recordings of the interviews were transcribed verbatim and then translated into English by the data collection team. The translated transcripts were read through several times to become familiar with the data and to get

an understanding of the participant’s accounts. The first author developed the initial coding scheme based on our objective, interview guide, and conceptual understanding of the referral system in Tanzania. To ensure reliability, two researchers coded the first transcript independently and then compared the codes for agreement on the final codes and coding. The coding was done manually. A list of potential and initial codes reflecting the predetermined themes was created through data reduction. Then sub-themes and themes were then generated from the general list of codes created through comparisons. Lastly, themes were reviewed and discussed by all authors, discrepancies were noted, and identified themes were finalized.

Ethical approval and consent to participate

Ethical clearance was obtained from the Muhimbili University of Health and Allied Sciences Institutional Review Board (MUHAS-REC-10-2021-867). The permission to conduct the study was obtained from the Director of Clinical Services at Amana Regional Referral Hospital and the Director of Clinical Services at Muhimbili National Hospital. Study participants were informed about study goals and objectives and were asked for permission to digitally audio-record. Information provided by the participant was kept confidential at all times and used for the research purpose. All the voices recorded were deleted after transcription and translation of the information. It was guaranteed that information was treated confidentially, anonymized before publication, and that quotes could not be traced back to individuals. We obtained written consent from all participants in the study.

Methodological considerations

Trustworthiness in a qualitative study is attained when the findings of such a study are worth believing [27]. Four criteria as explained by Shenton AK, et al. were used to assess the trustworthiness in our study; credibility, dependability, transferability, and conformability [28]. The credibility of findings was ensured through engagement and cross-checking of themes, sub-themes, and findings among research team members. In addition, during the data collection phase, the lead author spent a considerable amount of time interacting with study participants in both hospitals thus getting a better understanding of referral dynamics. The sharing of findings and the description of technical aspects of the qualitative methodologies used to ensure that our results are dependable. Out results are transferable to other contexts since the findings section provides thick descriptions of the procedures, settings, and methods and therefore lend

themselves relevant to other/similar contexts. Our study presents perspectives and experiences on referral from different cadres of staff in the two hospitals and therefore represents a fair range of differing viewpoints (authenticity) on issues around postpartum referrals in Tanzania.

The study achieved rich data through the involvement of maternity Health care providers with experience in the provision of care and referrals in the facilities under study. Furthermore, it provided an in-depth perspective of providers concerning intrapartum referrals thereby identifying gaps and areas for improvement. Despite all these strengths, the study had some limitations. A major limitation is the fact that an assessment of referral guidelines needed to be done to confirm the assertion of the adequacy of the guidelines. Therefore our results should be interpreted in light of this limitation.

Results

We interviewed 11 participants with an age range of 29-56 years. The average age of the participants was 40 years, where 45% were female and 55% were male. Among them, five were midwives, two registrars, three obstetrics and gynecologists, and one administrator. All of them had a working experience of at least three years.

Three main themes emerged from the analysis process each with 2 to 5 sub-themes: (1) Referral beyond medical indications (2) Limited maternity healthcare provider capability at referring facility (3) Limited communication between referring and receiving facilities. **See Table 2.**

Table 2: Themes and Sub-themes

**Themes**	**Sub-Themes**
Referral beyond medical indications	- Referral process<br>- Referrals due to a mismatch between delivery load and available facilities<br>- Referrals due to challenges in

	infrastructures<br>- Referrals to avoid blame<br>- Lack of motivation
Limited maternity healthcare provider capability at referring facility	- Lack of basic knowledge and skills in practices among maternity healthcare providers (MHP)<br>- The irresponsibility of MHPs at referring facility
Limited communication between referring and receiving facilities	- Harsh treatment to an escort nurse<br>- Lack of constructive feedback to the referring facilities

Theme 1. Referral beyond medical indications

This theme illustrates about experiences of participants on how the decision for referral is being made and other reasons for referrals. Five subthemes are described under this theme; the process of referral, referrals due to a mismatch between delivery load and available facilities, referrals due to challenges in infrastructures, referral to avoid blames, and lack of motivation.

The process of referral

The majority of referrals made were emergency intrapartum referrals. Before referral, standard operating procedures were not always followed. Participant stated that patients were referred without the required pre-referral management or appropriate documentation on the referral form. There was an increase in the number of referrals during the night compared to the day, and it was reported that poor supervision of junior doctors during the night affected decision- making and referrals at a late stage. A participant from receiving hospital was quoted saying,

"… A patient brought with an emergency condition having 18-20 gauges or resuscitated with 5 liters of fluid but came with no catheter and the patient is bleeding, you consider this as being lazy or they don't know their work…" (Administrator, receiving hospital)

It was responded with a participant from referring hospital was quoted saying,

"… sometimes we receive many patients at once who need to be referred, so following SOPs before referral might be a difficulty, otherwise, referrals must be issued out by the doctor upon agreement, and a referral form will be signed" (Overall in-charge, referring hospital).

Referrals due to a mismatch between delivery load and available facilities

It was believed by participants that the overwhelming number of patients at referring facilities influenced the decision to provide referrals. The hospital's infrastructure, including its operating rooms, neonatal and maternal intensive care units, and blood and transfusion product availability, precludes it from functioning as a referral hospital, which discourages it from providing referrals as a means of receiving assistance. A participant reported,

"… Right now, we have a big number of patients that we receive who are real emergencies but the environment itself in this hospital is not suitable to receive all of them…" (Specialist, referring hospital)

Another participant was quoted saying,

"… a patient referred from Mbagala Rangi Tatu hospital to Temeke hospital and straight was referred to Muhimbili hospital, that shows there was high a number of patients at Temeke that they couldn't manage that patient from Mbagala…" (Midwife, receiving hospital).

Referrals due to challenges in infrastructure

There was an unavailability of infrastructure such as intensive care units, high dependency units, enough operating rooms, the blood supply for transfusion, and an ambulance. When more than one emergency arises, then referral had to be issued. In addition, because of the availability of one ambulance, then they preferred to call for a referral at Upanga (a nearby national hospital

branch) compared to Mloganzila (a distant national hospital branch) because of the distance factor. To drive this point home, a participant quoted saying,

> *"When I was on duty yesterday, there was an urgent need for treatment for both an ectopic patient and a PPH patient, so I was compelled to take the PPH patient into the operating room while sending the Ectopic patient to a different hospital because there was no space to serve both patients"* (Registrar, referring hospital).

Another participant from receiving hospital said,

> *"A referral made early for a woman with fetal distress or two prior scars in labor, but the patient is brought 6 or 8 hours later, only to find out about the difficulties in finding an ambulance or an escort nurse who must cycle between more than two hospitals." (Midwife, receiving hospital)*

Referrals to avoid blame

When a patient or patient's relative is a member of the medical profession or has government influence, they frequently demand a referral for no valid reason. The majority of these obstacles are faced by hospital administrators who must endure the burden of receiving phone calls from patients demanding a referral.

> *"…But when a relative wants or is in favor of getting a referral to her relative and then you decide to retain a patient, when things go wrong, you can take the blame" (Administrator, referring hospital).*

Another participant added by saying, *"…we sometimes give a referral to the patient whom we could manage here, only to avoid delays and blames…" (Registrar, referring hospital.*

Lack of motivation

It was reported when providers did not receive their allowance payments on time, their motivation decreased, resulting in sub-optimal performance. Some doctors had a decreased desire to manage patients who visited a referring hospital during their shift. So that he/she could rest, he/she would choose to see some of the patients and refer other patients. Either participant reported that other doctors routinely disregard SOPs and refer patients without a genuine reason. A participant mentioned,

> *"We receive some referrals without an informing call and no convincing reason of referral, some doctors will just refer the patient knowing when the patient reached receiving hospital will never be rejected and taken back to our hospital" (registrar, receiving hospital).*

Theme 2: Limited maternity healthcare provider capability at referring facility

This theme illustrates the perception of MHPs' ability in the referring hospital. Two subthemes were identified: lack of basic knowledge and skills MHPs and irresponsibility in treating patients.

Lack of basic knowledge and skills in practices among MHPs at referring facility

Inadequate knowledge and skills among maternal care providers were found to exist, and some were incompetent in performing their responsibilities. Participants reported that when junior doctors failed to consult their seniors, ended up making wrong decisions that consequently caused delays in the provision of management to the patient.

> *"... Most of the time there is a gap, you find an intern doctor can't decide and still does not consult. At the end, decisions for referral are made very late" (Specialist, referring hospital)*

A participant from receiving hospital was quoted saying, *"...as I was working in the labor ward, there was a patient referred with a diagnosis of a previous scar with impending rupture, but after our investigation she was found to have umbilical hernia..." (Midwife, receiving hospital)*

The irresponsibility of MHPs at referring facility

Participants at the receiving hospital perceived the MHPs at the referring hospital as irresponsible since they sometimes refer patients unnecessarily. They further expressed that, sometimes upon phone calls they mention to refer two or three patients but they end up bringing four to five patients because they want to lessen the workload. One participant said:

> *" ... maybe they receive many patients, and they fail to attend all of them. You find there is no genuine reason to refer them. They call for referral of two patients and they bring 4 of them..." (Overall in-charge, receiving hospital).*

Theme 3. Limited communication between referring and receiving facilities

This theme illustrates the communication challenges among MHPs from the referring and receiving hospitals. These include harsh treatment of the MHPs by the referring hospital and lacking constructive feedback from the receiving hospital to the referring hospital.

Harsh treatment to an escort nurse

Participants state that an escort nurse who arrived with the patient at receiving hospital sometimes was less informed about the condition of the patient and the referral form was not filled properly. The predicament led the maternity staff at the receiving hospital to act hostilely against the escort nurses by presuming they would not do their duties as requested. A participant reported,

> *" ... I was called to escort the patient while I was in another ward, it was hard to know all the patient's information, especially if there were many patients. When you reached there, the staff acted haughtily and asked questions as if we didn't want to fulfill our obligations, even though that is not the case…" (Midwife, referring hospital).*

Lack of constructive feedback to the referring facilities

Participants reported that feedback from the receiving hospital was only sometimes given. Sometimes it was given as a report which tends to end up at the administration level and not reach the Maternity healthcare providers. For mismanaged patients who ended up in maternal death, feedback was given through phone calls.

> *"I was thinking there should be a procedure of sending feedback to the maternal care providers on the patients they referred and their outcome, we do write weekly reports, but it ended up at the managerial level and not reaching to the providers" (Midwife, receiving hospital)*

When a woman with an intrapartum emergency was referred to a tertiary hospital, she was received at the labor ward, where initial management, including resuscitations, was initiated. The on-call medical team reassesses and decides on patient disposal: whether she needs further consultations with senior doctors as inter-departmental consultations or surgical management within the maternity department. Unfortunately, no feedback information was sent back to the referring hospital regarding the progress of the management of the patient. A participant from referring hospital had this to say, "*…They hardly ever bring us feedback or let us, the providers, receive it. Feedback rarely reaches the service providers …" (Registrar, referring hospital)*

Discussion

This study explored the perceptions and experiences of maternity care providers regarding intrapartum emergency referrals in a regional referral hospital and Muhimbili National Hospital in Dar es Salaam, Tanzania. In response to our in-depth interviews, we found out that referrals beyond medical indications, such as referring a patient to avoid blame and deaths occurring at a particular health facility, limited competencies and irresponsibility of maternity healthcare providers, and limited communication between a referring and receiving health facility such as lacking constructive feedback and use of harsh language to escort health personnel were found to be the main findings.

Our study found that decision-making for the provision of referrals were influenced by high patient demand for quality service delivery. When a patient was health personnel or had an influence from the maternal care providers or from the government to obtain a referral without

an apparent reason. Since everyone wishes to receive the best care and the majority of patients believe the national hospital provides the best care, they demand referrals even without a genuine reason. Studies conducted in Indonesia and Tanzania [9, 11] found that providers perceived patients to be overly demanding during the referral process with the pressure they exerted on the delivery of services. In addition, the patient’s perceptions of the hospital's services place them under duress and have an effect on the referrals made. The relationship between the patient and provider is crucial to the delivery of quality services.

Intrapartum is a very crucial period that requires close care and monitoring to ensure the safety of both the mother and the baby. To meet patient demands and prevent adverse outcomes, maternal care providers often refer patients who need an apparent reason to avoid blame and delays and sometimes make referrals without communication with the receiving facility beforehand[11]. Similar findings on the referral to avoid delays and blames were found in a Sierra Leone study [12], which showed that some referrals were made to avoid complications or maternal death at their facility. Maternal care providers may refer a patient to avoid liability in the event of adverse outcomes or after receiving negative feedback. This could be explained by fear of damaging their reputation as MHPs and hospitals if a negative outcome happened.

The unfriendly working environment which lacked intensive care units and high dependency units for adults and neonates, a sufficient blood supply for transfusion, a sufficient number of operating rooms, a sufficient number of staffs, and timely payment of allowances lowered their motivation at work. Similar studies report staff shortage as a significant challenge influencing referrals [11, 16, 22]. Studies done in Ghana, Ethiopia and Indonesia [11, 16, 23] revealed similar findings regarding the need for more sufficient infrastructure and staff, which contributing to patient referral. The unconducive working environment contributed significantly to stress, frustrations, fatigue, low job satisfaction, and loss of morale. When providers are not paid their allowances on time, their morale to work at a high level is diminished. This could be because the government has upgraded these hospitals to referral hospitals, where the numbers of patients and workload have increased, but the working environment and staffing levels remained the same. It brought the challenge of increasing the number of referrals to the tertiary hospitals. This reduces the quality of services provided at tertiary hospitals because of needing more support.

In our study, some providers needed to gain skills in managing patients. These providers, who may be newly employed, interns, or those who were resistant to learning, caused delays in providing proper management or incorrect referral decisions. This could be explained by the need for more lack of adequate supervision from their seniors, who were not available at night. Similar studies reported that inadequate training, which leads to a lack of skills and inadequate compensation facilitates maternal care providers to refer patients to another facility [18]. Other studies [12, 24] showed that low quality within their designated skill set made influenced providers in referring women with intrapartum emergencies. Some colleagues were incompetent in making patients' diagnosis because he/she had little work experience compared to the senior provider; consequently, they tend to refer more patients due to a lack of skills and confusion regarding clinical referral criteria [23, 25]. The similarity may be due to the lack of motivation of the seniors for not being paid their allowances timely, so they opt to work during the day time and use other time for part time jobs to supplement their income.

Feedback provides an opportunity for providers to learn where they did wrong or the proper way to handle various cases, thereby improving the quality of services. According to our study, feedback was only sometimes provided, and there was no formal way of providing feedback. This could be a weakness on the part of the administration, wherein responsible personnel failed to perform their duties or raise a challenge if they had any, regarding the delivery of feedback. Studies highlighted the lack of regular feedback to referring facilities and inconsistent use of feedback forms, instead, only complaints about what needed to be appropriately done or expected to be done were reported [14, 26]. Other studies with comparable findings on referral feedback demonstrated that referring facilities were not always provided with feedback, and that feedback was sometimes given to the women or other close family members [6]. The lack of standard national referral guidelines had made no uniformity in delivering services, especially in giving feedback which is a barrier to a functional referral system [15].

Conclusion

Our study found that there was referral hostility between referring and receiving hospitals. The skills gap among maternal care providers at referring hospitals still existed which influencing the decision- making and provision of quality services. Referral should be regarded as the last option

after the patient has been managed to the best of knowledge at referring hospital, and pre-referral management should be provided before referral. Strategies to strengthen communication between the hospitals are warranted. These include ongoing training should be emphasized. Standard referral guidelines should be developed so that each hospital would know their roles and expectations. Further studies on the experiences of women and their relatives on referral services should be done, to get a clear picture of where we are lacking and what needs to be done.

Acknowledgments

The authors acknowledge the support received from all the participants in this study. We also thank Amana Regional Referral Hospital and Muhimbili National Hospital for granting access to use their facilities in this study.

Authors' contributions

SS conceptualized the study, conducted data collection, and analysis, and wrote the original draft. ABP and MME aided the conceptualization and analysis and supervised the writing. DLM and FAA participated in the analysis, writing, and reviewing the manuscript. MOM and SB edited and reviewed the manuscript. All authors reviewed and approved the final manuscript.

Conflict of interest

The authors declare that they have no conflicting interests.

Data availability

Data cannot be shared publicly because they contain sensitive participants' information and participants did not consent to having their interviews shared. Data are available from the Directorate of Research and Publications at the Muhimbili University of Health and Allied Sciences (contact via drp@muhas.ac.tz) for researchers who meet the criteria for access to confidential data.

Funding

The author (s) received no specific funding for this work.

References

1. WHO. Quality, Equity, Dignity: A Network for Improving Quality of Care for Maternal, Newborn and Child Health. 2017; February:14–6.

2. TDHS. Tanzania Demographic and Health Survey Indicator Survey (TDHS-MIS) 2015-2016. Dar es Salaam, Tanzania, Rockville, Maryland, USA MoHCDGEC, MoH, NBS, OCGS, ICF. 2016;1:1–630.

3. United Nations. The 2030 Agenda for Sustainable Development's 17 Sustainable Development Goals (SDGs). 2020.

4. Pembe AB, Urassa DP, Darf E, Carlsted A, Olsson P. Qualitative study on maternal referrals in rural Tanzania: decision making and acceptance of referral advice. Afr J Reprod Health. 2008;12:120–31.

5. Awoonor-Williams JK, Bailey PE, Yeji F, Adongo AE, Baffoe P, Williams A, et al. Conducting an audit to improve the facilitation of emergency maternal and newborn referrals in northern Ghana. Glob Public Health. 2015;10:1118–33.

6. Ameyaw EK, Njue C, Tran NT, Dawson A. Quality and women's satisfaction with maternal referral practices in sub-Saharan African low and lower-middle income countries: a systematic review. BMC Pregnancy Childbirth. 2020;20:1–16.

7. Ofosu B, Ofori D, Ntumy M, Asah-Opoku K, Boafor T. Assessing the functionality of an

emergency obstetric referral system and continuum of care among public healthcare facilities in a low resource setting: an application of process mapping approach. BMC Health Serv Res. 2021;21:1–14.

8. Simba DO, Mbembati NAA, Museru LM, Lema LEK. Referral pattern of patients received at the national referral hospital: challenges in low- income countries. East Afr J Public Health. 2008;5:6–9.

9. Proos R, Mathéron H, Vas Nunes J, Falama A, Sery Kamal P, Grobusch MP, et al. Perspectives of health workers on the referral of women with obstetric complications: A qualitative study in rural Sierra Leone. BMJ Open. 2020;10:1–8.

10. Benson AE, Benson MJ, Luke AH. Assessment of maternal referral systems used for a rural Zambian hospital: The development of setting specific protocols for the identification of complications. Afr Health Sci. 2019;19:1536–43.

11. Diba F, Ichsan I, Muhsin M, Marthoenis M, Sofyan H, Andalas M, et al. Healthcare providers' perception of the referral system in maternal care facilities in Aceh, Indonesia: A cross-sectional study. BMJ Open. 2019;9.

12. Afari H, Hirschhorn LR, Michaelis A, Barker P, Sodzi-Tettey S. Quality improvement in emergency obstetric referrals: Qualitative study of provider perspectives in Assin North district, Ghana. BMJ Open. 2014;4.

13. Austin A, Gulema H, Belizan M, Colaci DS, Kendall T, Tebeka M, et al. Barriers to providing quality emergency obstetric care in Addis Ababa, Ethiopia: Healthcare providers' perspectives on training, referrals and supervision, a mixed methods study. BMC Pregnancy Childbirth. 2015;15:1–10.

14. Daniels AA, Abuosi A. Improving emergency obstetric referral systems in low and middle-income countries: A qualitative study in a tertiary health facility in Ghana. BMC Health Serv Res. 2020;20:1–10.

15. Give C, Ndima S, Steege R, Ormel H, Mccollum R, Theobald S, et al. Strengthening referral systems in community health programs : a qualitative study in two rural districts of Maputo

Province, Mozambique. 2019;:1–11.

16. Pembe AB, Carlstedt A, Urassa DP, Lindmark G, Nyström L, Darj E. Effectiveness of maternal referral system in a rural setting: A case study from Rufiji district, Tanzania. BMC Health Serv Res. 2010;10.

17. Stal KB, Pallangyo P, Elteren M Van, Akker T Van Den, Roosmalen J Van. Women s' perceptions of the quality of emergency obstetric care in a referral hospital in rural Tanzania. 2015;20:934–40.

18. Mannava P, Durrant K, Fisher J, Chersich M, Luchters S. Attitudes and behaviors of maternal health care providers in interactions with clients: A systematic review. Global Health. 2015;11:1–17.

19. MOHSW T. Policy - National Health Policy. Minist Heal community Dev gender, elderly, Child Tanzania. 2017; October:1–55.

20. NBST. Sensa ya Watu na Makazi ya Mwaka 2022: Matokeo ya Mwanzo. 2022.

21. Clarke V, Braun V. Teaching thematic analysis : Overcoming challenges and developing strategies for effective learning Associate Professor in Sexuality Studies Department of Psychology Faculty of Health and Life Sciences University of the West of England Coldharbour Lane Br. Univ West Engl. 2013;26:120–3.

22. Chodzaza E, Bultemeier K. Service providers' perception of the quality of emergency obstetric care provided and factors identified which affect the provision of quality care. Malawi Med J. 2010;22:104–11.

23. Yasin C, Geleto A, Berhane Y. Referral linkage among public health facilities in Ethiopia: A qualitative explanatory study of facilitators and barriers for emergency obstetric referral in Addis Ababa city administration. Midwifery. 2019;79.

24. Ankita Kumari, Mitra S, Vani Aditya. The spectrum of obstetric referral and their outcome at a Tertiary Care Center of Eastern Uttar Pradesh: An insight. Asian J Med Sci. 2022;13:123–8.

25. Singh S, Doyle P, Campbell OM, Mathew M, Murthy GVS. Referrals between public sector health institutions for women with obstetric high risk, complications, or emergencies in India - A systematic review. PLoS One. 2016;11:1–23.

26. Bugomola MA. Effectiveness of Maternal Referral System in Tanzania : A Mixed Method Study. 2022;:1–18.

27. Graneheim UH, Lundman B. Qualitative content analysis in nursing research: Concepts, procedures and measures to achieve trustworthiness. Nurse Educ Today. 2004;24:105–12.

28. Shenton AK. Strategies for Ensuring Trustworthiness in Qualitative Research Projects Strategies for ensuring trustworthiness in qualitative research projects. 2016; February. https://doi.org/10.3233/EFI-2004-22201.
