# Supplementary material for: “Are all referrals necessary?” Experiences and perceptions of maternity healthcare providers on emergency intrapartum referrals in Dar es Salaam, Tanzania
Source: PLoS One. 2024 Feb 21;19(2):e0298103. doi: 10.1371/journal.pone.0298103 (PMC10880996; doi:10.1371/journal.pone.0298103)
Supplement: Attachment — Submitted filename: RESPONSE TO REVIEWERS.docx [file pone.0298103.s003.docx]

| **Reviewer’s comment** | **Author’s response** | **Page and lines the changes inserted** |
| --- | --- | --- |
| **Abstract** | | |
| Reviewer#1  The abstract is well developed and easy to understand | Thank you for the compliment | N/A |
| Reviewer 2  1. Not clear how you ensured the maximum variation sample eg. What were some of  The indicators of variation?  2. Why semi structured interview guide and not in-depth interview guide since your  aim was to explore the experiences and perceptions of maternity healthcare  Providers on emergency intrapartum referrals.  3. Compare data collection tool ‘semi-structured interview guide’ with what was  Stated in the materials and methods section ‘in-depth interviews.  4. The words: experiences and perceptions could be added to the key words. | The purposive sampling technique ensured maximum variation of participants by recruiting participants based on the expected reasonable coverage of the participants given the purpose of the study and interest. The indicators of variation included the differences in years of experience, cadre, levels, and study sites.  In-depth interview guide was used to explore perceptions and experiences of maternity healthcare providers on emergency intrapartum referrals. The changes have been made accordingly.  Thank you for your observation.  The changes have been made, now it reads as in-depth interview guide.  Thank you for the suggestion.  The two words have been added as suggested. | Page 7  Line 151-153  Page 8  Line 165  Page 2  Line 49-50 |
| **Introduction** | | |
| Reviewer 2  The study provided adequate introduction to the study | Thank you for the compliment. | N/A |
| **Conceptual framework** | | |
| Reviewer#1  This section is well discussed | Thank you | N/A |
| Reviewer 2  Under fig 2. The statement “the illustration above shows constructs that may influence  perception and experience of maternity care providers on emergency intrapartum referrals “ this  connotes that a figure could be referred to above, if statements are not following the fig.  Directly, then rephrase the statement to exclude the word ‘above’ | Thank you for your observation.  The word “above” has been removed. | Page 4  Line 93 |
| **Materials and methods** | | |
| **Study design and study setting**  Reviewer#1  The study settings have similarities in what? ,to complete the statement | Thank you for your observation.  The statement has been completed. | Page 5  Line 122-124 |
| **Study participants and recruitment**  Reviewer#1  Indicate number of each category of participants | The number of participants in each category have been indicated as suggested. | Page 7  Line 159-160 |
| **Data collection**  Reviewer#1  The segment is well written | Thank you. | N/A |
| Reviewer 2  Braun and Clarke thematic analysis involves six steps as you mentioned. If this approach  was used, let it be reflected in your analysis section the main steps Braun& Clarke  describes in their thematic analysis- check the use of code book with Braun and Clarke  esp. If a code book was used, then check your analysis steps as opposed to Braun and  Clarke’s approach. | Thank you for your comment.  Authors acknowledge to have developed and used a codebook. The analysis section has been revised accordingly to incorporate the missing steps based on the Braun and Clarke’s analysis approach. | Page 8,9  Line 182-194 |
| **Trustworthiness** | | |
| Reviewer#2  ‘The study achieved rich data by involving majority of maternity HCPs involved in  provision of  maternal care and referrals in the facilities that enabled giving a clear picture of what is  happening in referral system between referring facilities.’  I do not agree with this statement, selecting 11 participants from -84 referring facility and  197 staff of referral centre cannot be described as majority.  Pg 18 line ...7 check whether YOU MEANT ‘Thick’ or ‘Think’ descriptions.  Comparing with the main aim of the study, I do not think the statement below should be a  limitation, as your study sought to explore the perceptions and experiences of maternity  care providers.  ‘despite all these strengths, the study had some limitations. Exploring only perceptions and  experiences of maternal care providers narrowed the extend of data to be gathered as there  are many other angles related to emergency intrapartum referrals which could have been  left out related to mothers and their relatives’ views on the referrals ‘  I rather think the limitation could be the fact that you could have assessed or evaluated the  referral guidelines to confirm your assertion of inadequate guidelines. To complete your  story of intrapartum referrals. This could be explored further. | Thank you.  The statement has been re-phrased and the word ‘majority’ has been omitted.  Thank you.  Word has been changed to thick.  Thank you.  The limitation has been changed. Now the limitation statement reads as Assessment of referral guidelines was not done to confirm the assertion on adequacy of the guidelines” | **Page 10**  **Line 220-221**  Page 9  Line 216  Page 10  Line 224-226 |
|  |  |  |
| **Results** | | |
| Reviewer#1  Double check and write only all participants' quotes of 40 words and above in a block and maintain quotes less than 40 words in-text | Thank you.  All the quotes have been double checked and revised accordingly. | Page 11-16 |
| Reviewer#2  1. Provide a brief introduction of the major themes and how the subthemes are  interwoven before you begin the details about the subthemes.  2. ‘Mcp in receiving facilities perceive mcp in referring facility as incompetent and  Irresponsible’- this was a subtheme under the main theme “limited communication  between referring and receiving facilities communication” but I think it could have been a  major theme on its own.  3. Results section was characterised with grammatical issues, it was not clear  sometimes what the researcher was trying to communicate.  4. Most statements were winding and unclear.  5. What did you mean by ‘some had negative perceptions towards performing their  responsibilities’.  “participants reported that, feedback from the receiving hospital was not always given.  Sometimes it was given in a form of report which tends tend up to the level of  administration and  Not reaching to the maternal care providers” ?? please check the highlighted text for  repeated word. | Brief introductions have been made to all the major themes  Thank you for your suggestion. Authors have revised the results section and added one theme as suggested. Now, the number of themes has changed from 2 to 3 and all necessary changes have been made throughout the manuscript.  Grammatical issues on results section have been corrected.  Repeated word has been deleted. | Page 11-16  Page 11-16  Page 11-16 |
| **Discussion** | | |
| Reviewer#2  Below is a typical example of a winding statement:  2. ‘a study in Ghana, Ethiopia and Indonesia [11, 16, 23], also showed similar findings  on Unavailability of sufficient infrastructure and number of staffs where referrals  contributed by Deficiencies in working condition and environment of maternal care  providers contributed a lot of stress, frustrations, fatigue and poor job satisfactions  and loss of morally.’ ???? Reframe the above sentence.  3. These winding statements made it difficult to follow the discussions. A thorough  proof- reading of the entire manuscript is required.  4. ‘lack of proper referral guidelines had made no uniformity in delivering of services  Especially giving feedbacks which are a barrier to a functional referral system [15].’  5. What is the meaning of the above statement? is there a standardised national  protocol on referral systems, or there is none? | Thank you for noting this.  The sentence has been reframed accordingly.  Proof-reading of the entire manuscript has been done as suggested.  Thank you.  There is no standardized national referral systems as well as the guideline for referrals. | Page 17  Line 402-404  Page 16-18  Full lengthy manuscript. |
| Conclusion  Reviewer 1  The manuscript is well-written and covers an interesting topic.  Having read and reviewed this manuscript, I am confident it can be accepted for publication after addressing some minor changes that have been suggested across the manuscript, including reviewing some quotation styles where quotations that are less than 40 words could be in quotation marks within the text and present all quotation of 40 or more words in a block quotation.  In addition, it is suggested that the section on methodological considerations should come before the section on results and findings. | Thank you for the compliment.  Methodological consideration segment has been moved to the methodology section just before the results section. | Page 9  Line 206-226 |
| Reviewer#2  The conclusion could have included development or review of a referral manual or  protocol, so that each party would be aware of their roles and expectations | Thank you for the suggestion.  The point has been added,  ‘Standard referral guideline should be developed, so that each hospital would be aware of their roles and expectations’ | Page 19  Line 443-445 |
